# DNA-targeting Invader probes: discovery, principles and applications

**DOI:** 10.1039/d5cb00286a

**Published:** 2026-01-23

**Authors:** Patrick J. Hrdlicka, Michaela E. Everly

**Affiliations:** a Department of Chemistry, University of Idaho Moscow Idaho 83844-2343 USA hrdlicka@uidaho.edu

## Abstract

Development of chemically modified oligonucleotides, nucleic acid mimics, protein-based constructs, and other ligands – capable of sequence-unrestricted recognition of specific double-stranded (ds) DNA regions – is an area of research that continues to attract considerable attention. Efforts are fueled by the need for diagnostic agents, modulators of gene expression, and novel therapeutic modalities against genetic diseases. While pioneering approaches focused on accessing nucleotide-specific features from the grooves of DNA duplexes, recent developments have entailed strand-invading probes, *i.e.*, probes capable of binding to DNA duplexes by breaking existing Watson–Crick base pairs and forming new, more stable base pairs. For the past twenty years, our laboratory has pursued the development of a type of dsDNA-targeting strand-invading probes, which we have named Invader probes. These double-stranded oligonucleotide probes feature intercalator-functionalized nucleotides that are specifically arranged to promote destabilization of the probe duplex, whereas individual strands exhibit very high affinity towards complementary DNA. This account details the discovery, principles, and applications of Invader probes.

## Abstract 2

Invader are double-stranded oligonucleotide probes with specific zippers of intercalator-functionalized nucleotides that enable efficient and highly specific recognition of complementary DNA regions *via* a strand-invasion mechanism.

## Graphical abstract

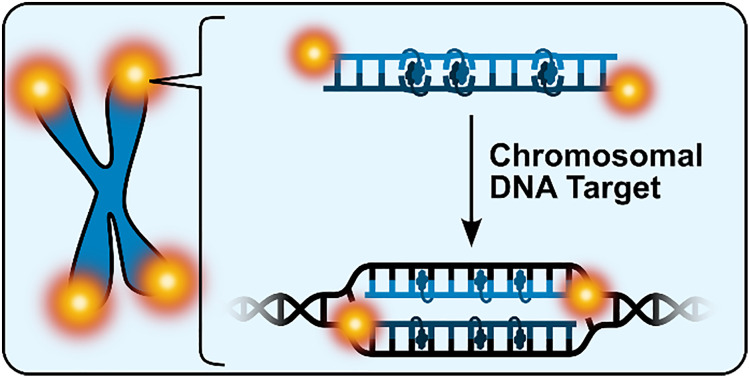

## Introduction

Over the past several decades, considerable efforts have been devoted to the development of synthetic probes capable of recognizing specific chromosomal DNA regions. These efforts have been fueled by the prospect of diagnostic agents, modulators of gene expression, and novel therapeutic modalities against genetic diseases. However, while more than twenty RNA-targeting antisense oligonucleotides (ONs), splice-switching ONs, and small interfering RNAs (siRNAs) have received regulatory approval in recent years as therapeutics against genetic diseases,^1^ DNA-targeting ONs have yet to achieve similar success. This reflects the additional challenges that DNA targeting poses, such as the need for nuclear delivery of DNA-targeting ONs and high compaction of chromosomal DNA. Moreover, the DNA duplex only offers few nucleotide-specific handles that can be accessed by synthetic ligands, as the most information-rich features, *i.e.*, the Watson–Crick base pairs, are buried deeply within the duplex core.

**Fig. 1:**
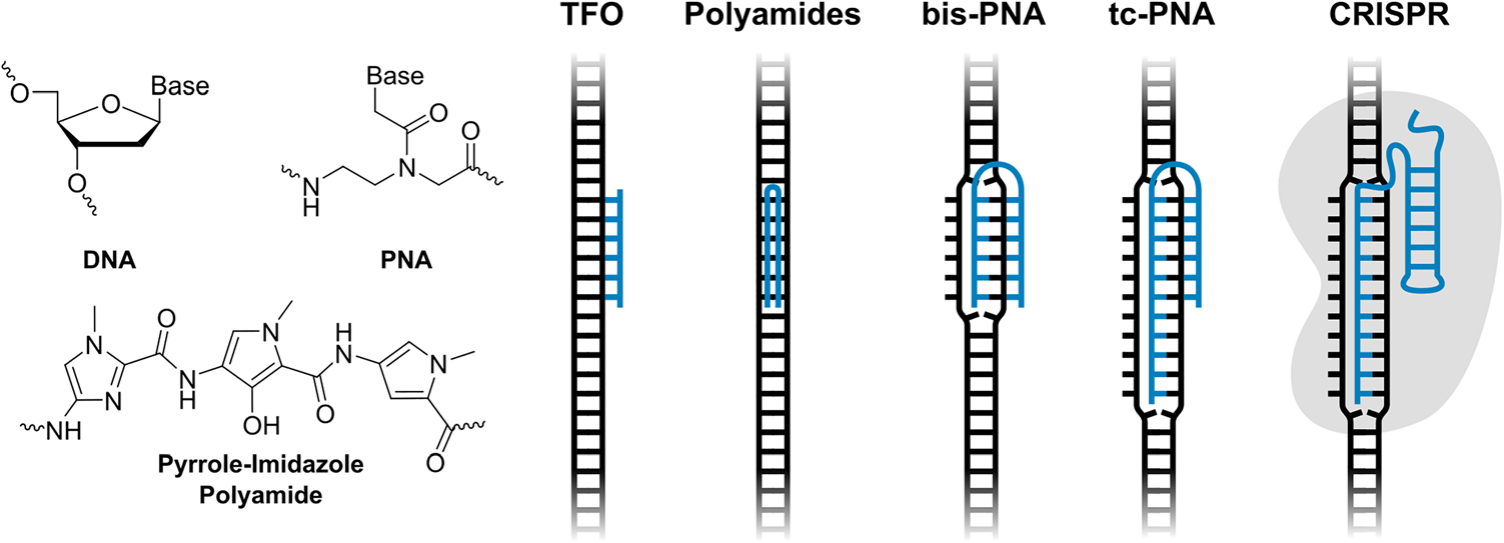
Overview of pioneering DNA-targeting strategies.

Pioneering probe technologies capable of targeting double-stranded DNA (dsDNA) include minor groove-binding pyrrole-imidazole polyamides,^2,3^ and major groove-binding triplex-forming ONs (TFOs)^4,5^ and peptide nucleic acids (PNAs),^5,6^ which access nucleotide-specific features from the duplex grooves (Fig. 1). However, these approaches have a limited target scope; polyamides are generally directed towards short dsDNA regions as binding- and shape-complementarity is compromised when longer regions are targeted, whereas stable triplex formation by TFOs and PNAs is normally restricted to regions with extended polypurine tracts. While polyamides can be linked to target longer dsDNA regions,^7^ and strategies reducing the polypurine requirement for triplex formation have been developed^5,8–13^ – such as alternate strand TFOs,^11,12^ the use of modified nucleotides capable of recognizing interruption sites,^10,13^ or joining two PNA strands *via* a non-targeting linker (bis-PNA)^8^ and extending the Watson–Crick binding end of the bis-PNA to yield tail-clamp (tc)-PNA^9^ (Fig. 1) – this increases the complexity of the probes without fully addressing the challenges.

Recently, CRISPR–Cas9 has received considerable attention for DNA-targeting applications (Fig. 1). While the potential of CRISPR–Cas9 and related approaches is clear, current limitations must be acknowledged; CRISPR-based systems often suffer from immunogenicity, delivery challenges and specificity concerns.^14^ Efforts to address these limitations are needed for CRISPR to become a universal tool for *in vivo* applications.

**Fig. 2:**
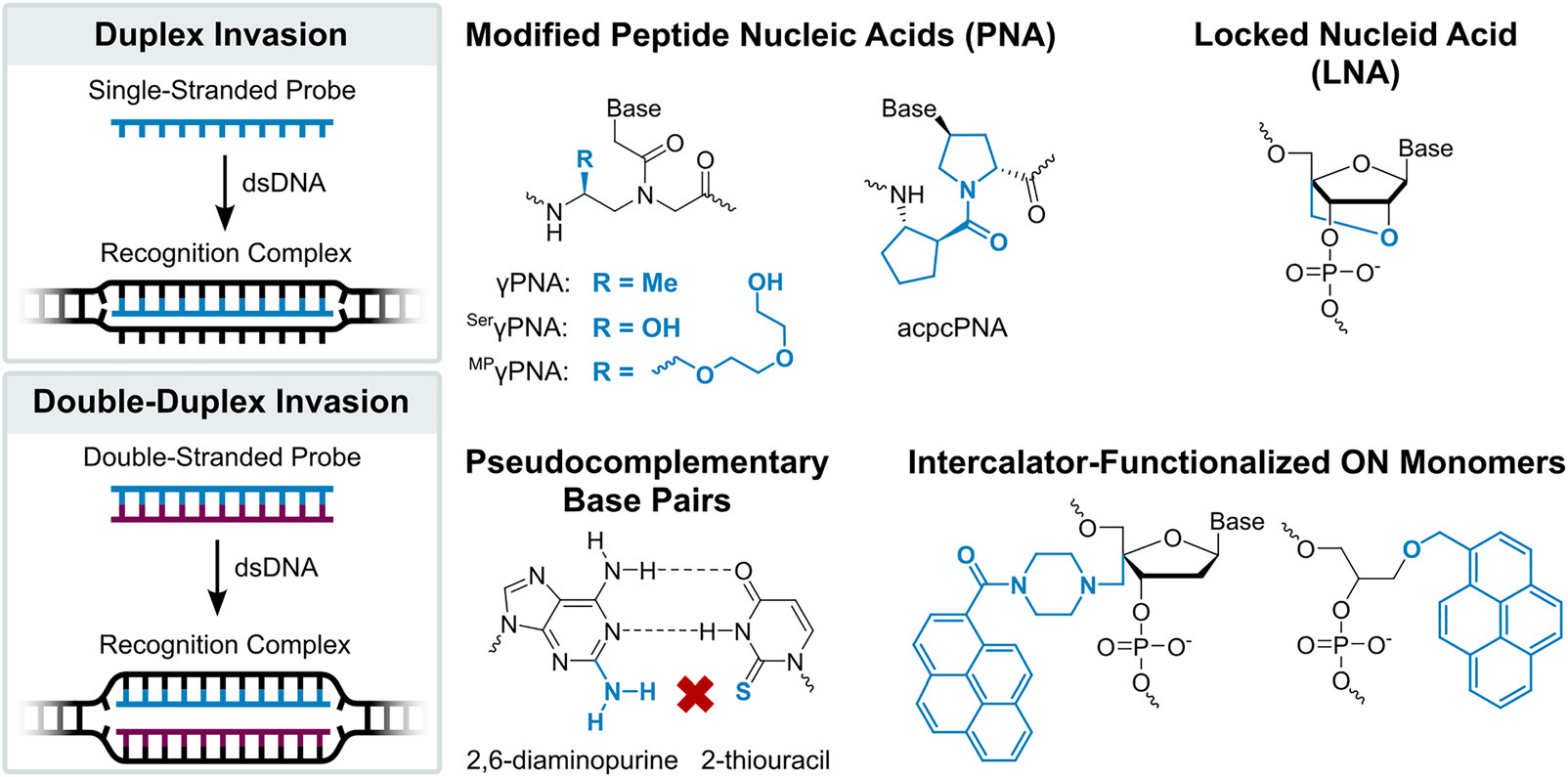
Illustrations of DNA recognition mechanisms and overview of chemistries used in strand-invading probes.

Development of simpler, ON-based probe technologies capable of sequence-unrestricted recognition of double-stranded DNA therefore remains an important endeavor. Towards this end, numerous strand-invading probes have been explored, *i.e.*, probes capable of breaking existing Watson–Crick base pairs of dsDNA regions to form new, more stable base pairs between probe strands and complementary DNA (cDNA) regions. Notable examples of strand-invading probes (Fig. 2) – among others^15–17^ – include:

– γPNA, in which the electrostatically neutral and achiral *N*-(2-aminoethyl)glycine backbone of PNA is additionally modified at the γ-position with a chirality-inducing moiety such as an (*R*)-methyl group (^Me^γPNA), resulting in preorganization of the strand and increased cDNA affinity compared to conventional PNA.^18^ To increase the aqueous solubility and reduce aggregation of γPNAs, (*R*)-hydroxyl (^Ser^γPNAs) or (*R*)-diethylene glycol moieties (mini-PEG (MP), ^MP^γPNAs) have been introduced at the γ-position.^19,20^

– acpcPNA, *i.e.*, single-stranded PNAs featuring an (2′*R*,4′*R*)-proline/(1*S*,2*S*)-2-aminocyclopentanecarboxylic (acpc) backbone. The pyrrolidine ring and β-amino acid of this α/β-dipeptide backbone preorganize the strand for efficient dsDNA invasion, while the inherent bulkiness of the acpcPNA moieties prevent self-hybridization.^21^

– Locked nucleic acids (LNAs), *i.e.*, single-stranded ONs that are extensively modified with 2′-*O*,4′-*C*-methylene-β-d-ribofuranosyl monomers. The dioxabicyclo[2.2.1]heptane skeleton locks the furanose ring in an *N*-type conformation (C3′-*endo*, pseudorotational phase angle *P* = 17°), leading to structural preorganization of the strand for target recognition.^22^ While primarily renowned for their RNA-binding properties,^23^ LNAs have been shown to recognize mixed-sequence dsDNA regions under conditions in which the targets are particularly accessible.^24–27^

– Pseudocomplementary DNA and PNA (pcDNA/pcPNA),^28–31^*i.e.*, short DNA or PNA duplexes that are modified with pseudocomplementary base pairs such as 2-thiouracil and 2,6-diaminopurine. Steric clashes between the pseudocomplementary base pairs perturb and weaken the hydrogen bonding between these moieties, resulting in destabilized probe duplexes, while the pseudocomplementary nucleobases form stable base pairs with canonical nucleobases. The stability difference between the double-stranded probes and the corresponding duplexes between individual probe strands and cDNA provides the driving force for double-duplex invasion of dsDNA target regions under certain conditions.

– Heteroduplexes between intercalator-modified ONs and complementary PNA, RNA, or LNA strands.^32–38^ These approaches rely on intercalators typically being accommodated less well in PNA/DNA duplexes or A-type (RNA-like) duplexes,^39,40^ than B-type (DNA-like) duplexes. As a result, the heteroduplexes are less stable than the corresponding duplexes between individual probes strands and cDNA. Akin to pcDNA and pcPNA, this stability difference provides the driving force for recognition of dsDNA targets.

A key advantage of strand-invading probes is that dsDNA regions of any sequence composition, in principle, can be targeted. However, single-stranded probes that are extensively modified with affinity-enhancing modications tend to self-hybridize,^35–38,41^ which can compromise target binding (acpcPNA being a notable exception).^21^ Double-stranded probes, in turn, must be engineered to denature easily whilst maintaining high cDNA affinity.

**Fig. 3:**
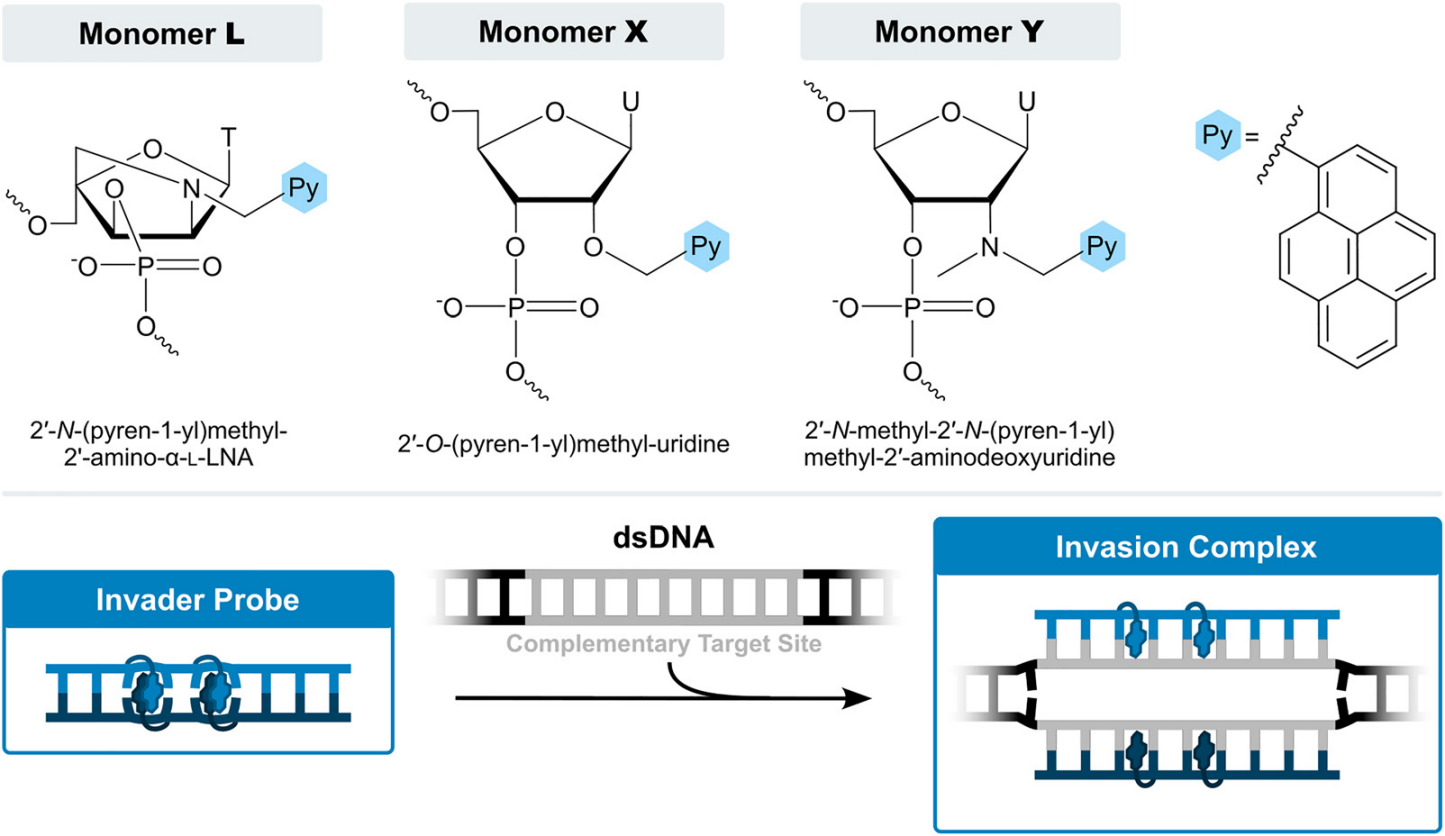
Structures of key Invader monomers and illustration of Invader probe-mediated invasion of dsDNA.

For the past twenty years, our laboratory has pursued development of an alternative class of dsDNA-targeting probes relying on double-duplex invasion, which we have named Invader probes. These are double-stranded oligonucleotide probes featuring intercalator-functionalized nucleotides that are specifically arranged to promote destabilization of the probe duplex, while individual strands exhibit very high affinity to cDNA (Fig. 3). This account will describe the discovery, principles, and applications of Invader probes.

## Discovery of the Invader principle

**Fig. 4:**
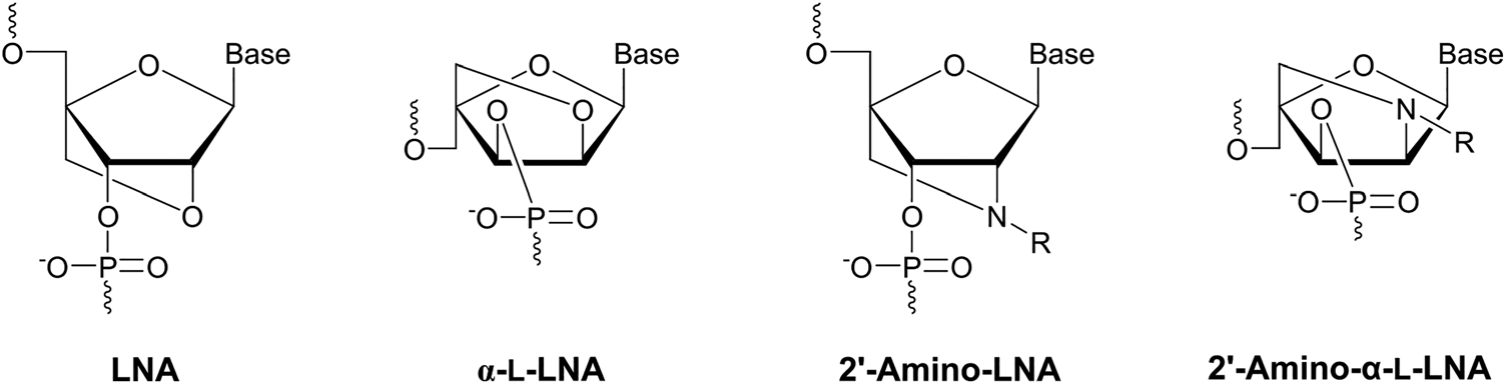
Structures of LNA, α-l-LNA, and N2′-functionalized 2′-amino-LNA and 2′-amino-α-l-LNA monomers.

Following its pioneering work on conformationally restricted and affinity-enhancing locked nucleic acid (LNA) and α-l-LNA nucleotide monomers in the late 1990s and early 2000s (Fig. 4),^42,43^ Prof. Jesper Wengel's laboratory developed an interest in using functionalized oligonucleotides for nucleic acid-based Ångstrøm-scale engineering.^44^ This, in turn, fueled an interest in N2′-functionalized derivatives of 2′-amino-LNA^45,46^ and 2′-amino-α-l-LNA monomers^47,48^ (Fig. 4) as substitution of the oxygen atom for a nitrogen atom allows for introduction of additional, precisely positioned moieties, such as fluorescent pyrenes.^49^ One of us (PJH) was charged with devising a synthesis of N2′-functionalized 2′-amino-α-l-LNA thymine monomers, after an initial attempt had unexpectedly yielded the corresponding 2′-amino-*xylo*-LNA monomers^50^ instead.

### Synthesizing N2′-pyrene-functionalized 2′-amino-α-l-LNA monomers

**Scheme 1:**
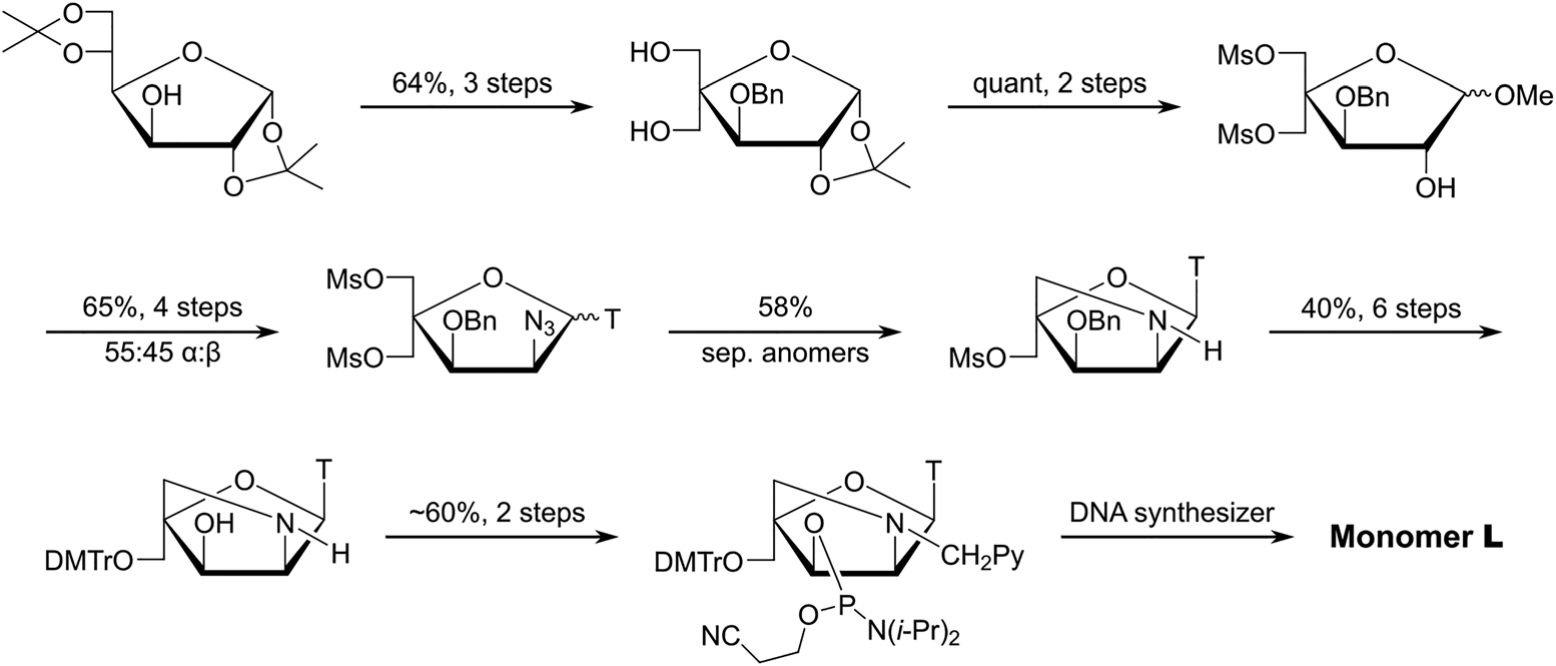
Outline of the synthetic route to the phosphoramidite of 2′-*N*-(pyren-1-yl)methyl-2′-amino-α-l-LNA thymine monomer L.

Following extensive experimentation, a long but viable synthetic route to the phosphoramidite of 2′-*N*-(pyren-1-yl)methyl-2′-amino-α-l-LNA thymine monomer L (*i.e.*, the first Invader building block, *vide infra*) was developed using inexpensive diacetone-α-d-glucose as a starting material (∼3% overall yield, 18 steps, Scheme 1).^51,52^ Key steps included: (i) introduction of a C2-azido group *prior* to installation of the nucleobase, (ii) Vorbrüggen glycosylation affording an inseparable anomeric mixture of α- and β-nucleosides (∼55 : 45 ratio), (iii) separation of subsequent α-l-*ribo*- and β-l-*ribo*-configured bicyclic nucleoside intermediates, (iv) selection of a suitable protecting group to prevent intramolecular Michael addition of the C2′-amino group to the C6-position of thymine and formation of tetracyclic 5,6-dihydrothymine derivatives, and (v) N2′-functionalization of the DMTr-protected key amino alcohol intermediate. Other intercalators were subsequently introduced in this manner.

**Scheme 2:**
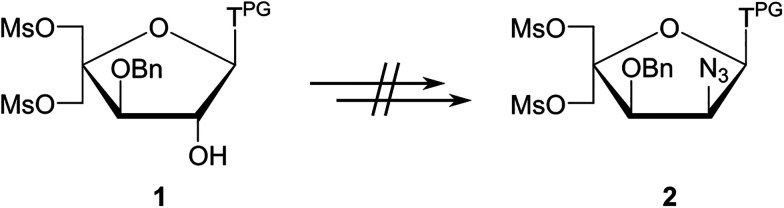
Unsuccessful attempts at shortening the route to key intermediate 2 in the synthesis of 2′-*N*-(pyren-1-yl)methyl-2′-amino-α-l-LNA thymine monomers.

Several attempts were made to shorten the route and avoid formation of anomeric nucleoside mixtures by converting nucleoside intermediates of the general type 1 into intermediate 2 (Scheme 2).^51^ However, these attempts were unsuccessful, either resulting in undesirable formation of the corresponding O2,O2′-anhydronucleosides (eventually leading to the undesirable downstream formation of 2′-amino-*xylo*-LNA nucleosides^50^) or no reaction (presumably due to the steric hindrance at the C2′(α)-position stemming from the 1,3-*cis* orientation of the thymine moiety and the C3′-benzyloxy group).

**Scheme 3:**
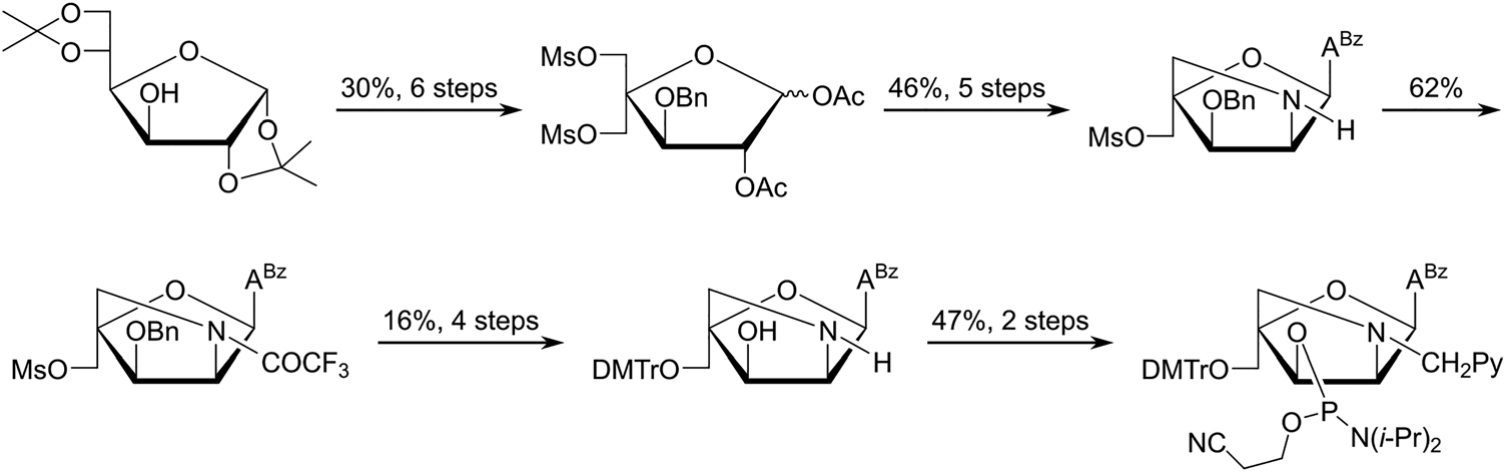
Outline of the synthetic route to the phosphoramidite of the adenine analogue of monomer L.

In parallel, we developed a synthetic route to the corresponding *N*6-benzoyladenine analogue of monomer L, but this proved even more challenging (<1% overall yield over 18 steps from diacetone-α-d-glucose, Scheme 3).^53^ Although the key C2′-azido group could be successfully introduced with anchimeric assistance from the O2′-acetyl group after installation of the nucleobase – thereby avoiding formation of anomeric nucleoside mixtures – the route was marred by challenging downstream protecting group manipulations, which reduced the overall yield. Nonetheless, enough amidite was obtained for preliminary characterization studies.

**Key point:** Synthetic routes to the corresponding phosphoramidites of 2′-*N*-(pyren-1-yl)methyl-2′-amino-α-l-LNA monomers have been developed. However, the routes are long and low-yielding.

### Preliminary characterization of ONs modified with 2′-*N*-(pyren-1-yl)methyl-2′-amino-α-l-LNA monomers

In an initial study, we were pleased to discover that 9-mer ONs modified with a single L monomer form exceptionally stable duplexes with cDNA as evidenced by increases in thermal denaturation temperatures (*T*_m_s) of up to +15.5 °C, relative to the corresponding unmodified DNA duplexes (*i.e.*, Δ*T*_m_).^54^ A subsequent study^52^ revealed that the extent of duplex stabilization significantly exceeds that of α-l-LNA-T or unfunctionalized 2′-amino-α-l-LNA-T monomers (Δ*T*_m_s = up to +8.0 °C and +2.5 °C, respectively), indicating that the pyrene moiety has a significant stabilizing role that acts in addition to any affinity-enhancing effects of the bicyclo[2.2.1]heptane scaffold. In stark contrast, incorporation of a single 2′-*N*-ethyl-2′-amino-α-l-LNA thymine monomer – *i.e.*, a *de facto* replacement of the pyrene moiety for a methyl group – results in dramatic destabilization of DNA duplexes (Δ*T*_m_s as low as −12.0 °C),^52^ further underscoring the stabilizing role of the pyrene moiety. Duplex stabilization has been attributed to the formation of strong π–π stacking interactions between the intercalating pyrene moiety and flanking base pairs, a binding hypothesis that is additionally supported by the following observations:^52,54^

– L-modified ONs display prominent DNA selectivity, *i.e.*, duplexes between L-modified ONs and complementary RNA (cRNA) are far less stabilized (Δ*T*_m_ (ON:cDNA) − Δ*T*_m_ (ON:cRNA) = 6.5–9.0 °C). Intercalators are known to favor B-type duplexes over A-type duplexes,^40^ as the former are more elongated, with more efficient base pair overlaps for stronger stacking interactions with intercalators. Accordingly, DNA selectivity is often observed for ONs modified with intercalating pyrene moieties.^32,55–58^

– Compared to unmodified ONs, L-modified ONs display reduced discrimination of DNA strands with mismatched nucleotides opposite the pyrene-modified monomer. Thus, duplexes between a centrally L-modified 9-mer ON and DNA strands with mismatched C/G/T residues opposite of the L monomer displayed Δ*T*_m_ values of −12.5/−5.5/−3.5 °C relative to the corresponding matched duplexes, respectively, as compared to Δ*T*_m_ values of −16.5/−9.5/−17.0 °C for the corresponding unmodified ON.^52^ This is likely because strong stacking interactions between the intercalating pyrene moiety and flanking mismatched base pairs compensate for the loss in stability from the distorted base pair.^59,60^

– Duplex formation between L-modified ONs and cDNA results in bathochromic and hypsochromic shifts of pyrene absorption maxima, which are indicative of strong electronic interactions between an intercalating pyrene moiety and flanking nucleobases.^61^

**Fig. 5:**
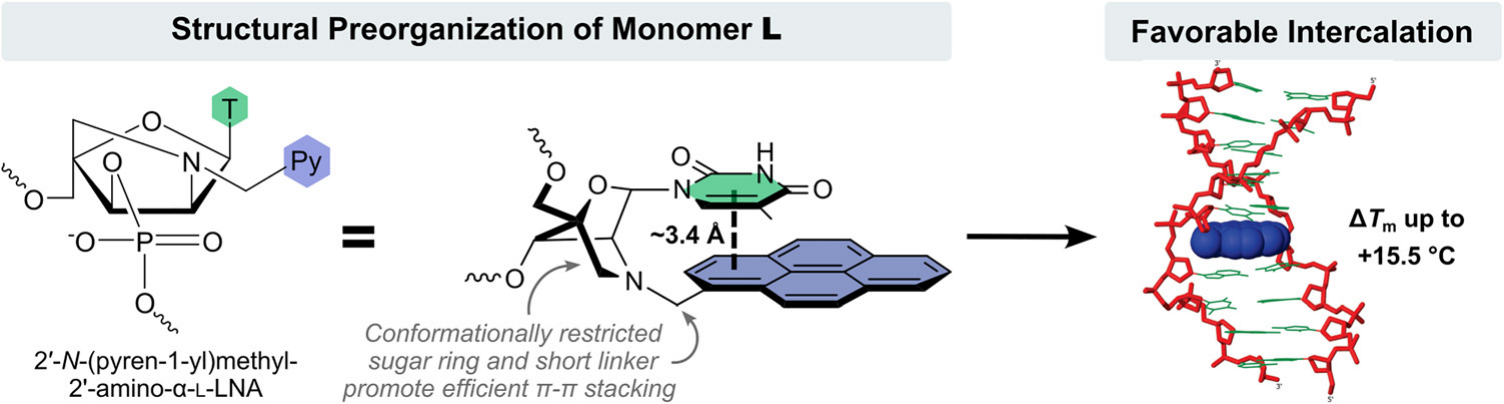
Structural preorganization of monomer L resulting in forced intercalation of the pyrene moiety. Image adapted from ref. 52 with permission from the American Chemical Society, copyright 2009.

Closer inspection of the structure of monomer L reveals that the attachment points of the nucleobase and pyrene moieties are restricted relative to each other due to the rigid nature of the 2-oxo-5-azabicyclo[2.2.1]heptane scaffold and the short methylene linker used to connect the pyrene. This promotes π–π stacking between the nucleobase and pyrene moiety, which accordingly is preorganized to intercalate. Molecular modelling studies provided further support for this hypothesis (Fig. 5). The lowest energy structure of a singly L-modified DNA duplex featured an intercalating pyrene moiety engaging in extensive π–π stacking with the thymine of monomer L and the 3′-flanking nucleobase and, to a lesser extent, the nucleobases on the complementary strand across of these moieties.^52^ Consistent with this, duplex stabilization is more pronounced when the L monomer is flanked by 3′-purines with larger π-stacking surfaces.^52,54^

**Key point:** ONs modified with 2′-*N*-(pyren-1-yl)methyl-2′-amino-α-l-LNA monomers exhibit exceptional cDNA affinity due to 3′-directed intercalation of the pyrene moiety.

### DNA duplexes modified with different interstrand zippers of 2′-*N*-(pyren-1-yl)methyl-2′-amino-α-l-LNA monomers

**Fig. 6:**
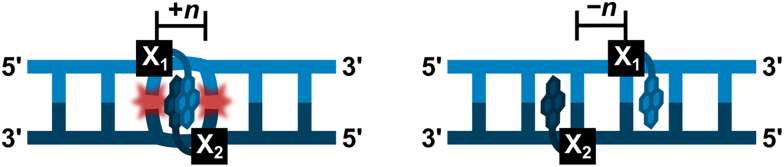
Illustration of interstrand zipper arrangements. The number *n* describes the distance measured in number of base pairs and has a positive value if a monomer is shifted toward the 5′-side of its own strand relative to a second reference monomer on the other strand or a negative value if a monomer is shifted toward the 3′-side of its own strand relative to a second reference monomer on the other strand.

After establishing the high cDNA-affinity of L-modified ONs, a series of 9-mer duplexes with different “interstrand zipper arrangements” of L monomers were evaluated (Fig. 6),^54^ to explore novel structural motifs potentially capable of interstrand communication for nucleic acid-based Ångstrøm-scale engineering.^44^

**Fig. 7:**
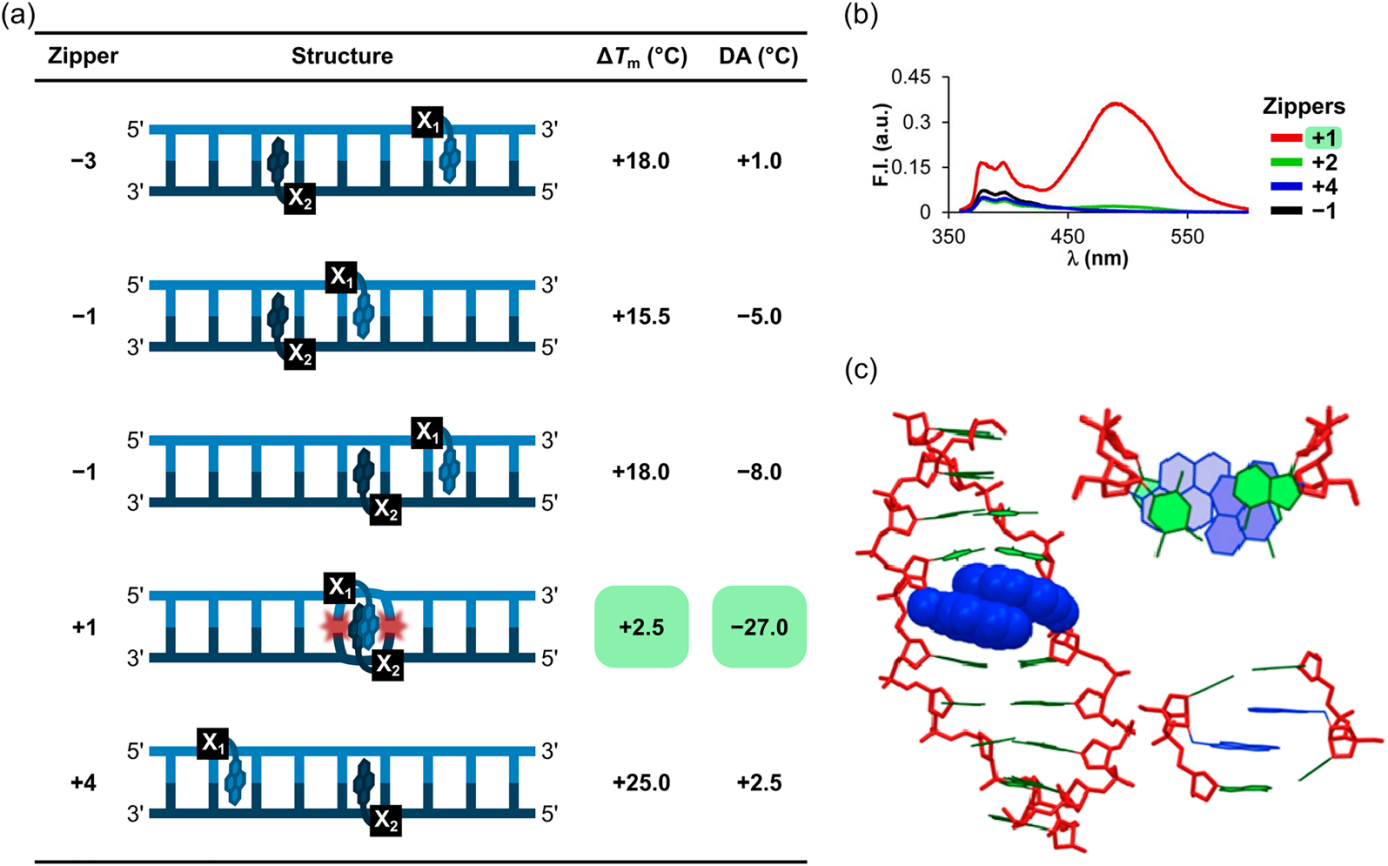
Characteristics of DNA duplexes modified with different interstrand zipper arrangements of L monomers. (a) Δ*T*_m_ and DA values. (b) Steady-state fluorescence emission spectra (*λ*_ex_ = 350 nm) of DNA duplexes with +1, +2, +4, or −1 zippers of L monomers. (c) Energy-minimized structure of a DNA duplex with a +1 zipper of L monomers. Figure adapted from ref. 62 with permission from the American Chemical Society, copyright 2013.

Duplexes with −3, −1, and +4 interstrand zipper arrangements of L monomers were found to be strongly stabilized, with Δ*T*_m_ values between +15.5 °C and +25.0 °C (Fig. 7a). To assess if two L monomers contribute independently to duplex stability, the term deviation from additivity (DA) was introduced and calculated as follows: DA_ONX:ONY_ = Δ*T*_m_(ONX:ONY) − Δ*T*_m_(ONX:cDNA) − Δ*T*_m_(ONY:cDNA), where ONX:ONY represents a duplex with an interstrand zipper arrangement of monomers.^62^ For duplexes with −3, −1, and +4 zippers of L monomers, the DA values ranged from −8.0 °C to +2.5 °C, indicating that the L monomers largely impact DNA duplex stability independently from each other with contributions ranging from moderately less-than-additive to slightly more-than-additive (Fig. 7a). Crucially, a DNA duplex with a +1 zipper of L monomers was found to be considerably less stable with a Δ*T*_m_ value of +2.5 °C and a DA value of −27.0 °C (Fig. 7a). The corresponding duplex with a +2 zipper of L monomers was also labile but exhibited less prominent destabilizing interference between the two L monomers (Δ*T*_m_ ∼ 0 °C, DA = −13.5 °C; Fig. 7a). Subsequent characterizations of DNA duplexes with a broader range of interstrand zipper arrangements of L monomers revealed that the prominent destabilizing interference is unique to +1 zippers.^63^ As will be discussed in subsequent sections, this is the key structural motif that forms the basis for dsDNA-targeting Invader probes.

DNA duplexes with +1 (or any other) interstrand zippers of α-l-LNA-T or unfunctionalized 2′-amino-α-l-LNA-T monomers do not display comparable destabilizing interference (shown later in Table 1), reinforcing the notion that the distinctive nature of DNA duplexes with +1 zippers of L monomers, primarily is due to the two pyrene moieties, rather than the proximity of two bicyclo[2.2.1]heptane scaffolds.^62^

The distinctive nature of DNA duplexes with +1 zippers of L monomers was further underscored by the presence of prominent pyrene excimer signals, centered at 495 nm, in the steady-state fluorescence emission spectra of these duplexes (Fig. 7b).^54,63^ These signals imply a coplanar arrangement of pyrene moieties with an interplanar separation of ∼3.4 Å.^64^ In contrast, duplexes with other interstrand zipper arrangements exhibited negligible, if any, emission in this region (Fig. 7b).

Preliminary force field simulations of a 9-mer DNA duplex with a +1 zipper of L monomers indicated that the spatial separation between the two pyrene moieties is too large to account for excimer formation across the major groove (estimated shortest distance ∼12 Å).^54^ Subsequent, more extensive molecular modeling studies suggested that simultaneous 3′-directed intercalation of both pyrene moieties is possible (Fig. 7c).^62^ The resulting stacking arrangement is consistent with excimer formation and perturbs neighboring base pairs and partially elongates and unwinds the probe duplex, consistent with reduced duplex stability. It should be noted that the lowest energy structure from this simulation only featured one intercalating pyrene, while the other pyrene projected into the major groove.^62^ While this structure also could explain the low duplex stability (*i.e.*, placement of a non-polar pyrene perturbing the hydration spine in the major groove), it does not account for the observed pyrene excimer emission.

These conclusions were additionally substantiated by NMR studies on a 13-mer DNA duplex with a central +1 zipper of L monomers. The ^1^H NMR signals from nucleotides proximal to the L monomers exhibited progressive broadening, while signals of protons close to the pyrene moieties were entirely absent. This indicates a significant level of perturbation and flexibility near the +1 zipper.^62^ Similar structural consequences were previously reported for NMR structures of DNA duplexes with adjacent incorporations of pyrene-modified non-nucleotide monomers.^65^

The perturbations in the vicinity of the +1 zipper of L monomers are consistent with a violation of the nearest-neighbor exclusion principle (NNEP).^66,67^ The principle posits that intercalator densities exceeding one intercalator per two base pairs are unfavorable in DNA duplexes. This is due to constraints in local helix expandability (each intercalation event expands the duplex by ∼3.4 Å) and because stabilizing stacking interactions between flanking base pairs and a first intercalating moiety are perturbed upon intercalation of a second moiety.^68,69^

Duplexes with +1 zippers of L monomers exhibit a local density of two intercalators per two base pairs – which represents a major violation of the NNEP – resulting in a labile duplex (*e.g.*, see +1 zipper structure in Fig. 7a). Duplexes with +2 zippers of L monomers or 0 zippers between the L monomer and its corresponding adenine derivative have a local density of two intercalators per three base pairs according to our binding model (*e.g.*, see the +2 zipper structure in Fig. 7a), which represents a more moderate violation of the NNEP, consistent with the less pronounced destabilizing interference.^54,62^ Duplexes with other interstrand zipper arrangements of L monomers are very stable since the NNEP is not violated (local intercalator density is – at most – one intercalator per two base pairs).^54,63^ Duplexes between ONs modified with interspersed L monomers and cDNA are also very stable since the local intercalator density never exceeds the NNEP limit.

**Key point:** DNA duplexes with a +1 zipper of L monomers are labile, presumably due to 3′-directed intercalation of both pyrene moieties into a shared region between neighboring base pairs, leading to unique destabilizing interference and prominent violation of the NNEP. Less prominent or no destabilizing interference between the pyrene moieties are observed in DNA duplexes with other interstrand zipper arrangements of L monomers and in duplexes between ONs with non-sequential incorporations of L monomers and cDNA.

### Defining key terminology

For the remainder of this account, the structural motif described as a +1 interstrand zipper of pyrene-functionalized nucleotide monomers (and more generally, a +1 interstrand zipper of intercalator-functionalized nucleotide monomers) is referred to as an *energetic hotspot*. An oligonucleotide-based duplex featuring one or more energetic hotspots, is referred to as an *Invader probe*. An Invader probe featuring one or more energetic hotspots of L monomers, is specifically referred to as an *Invader LNA probe*.

### Initial dsDNA-recognition experiments using double-stranded probes with +1 zippers of 2′-*N*-(pyren-1-yl)methyl-2′-amino-α-l-LNA monomers

The difference in stability between DNA duplexes with a +1 interstrand zipper of L monomers (*i.e.*, Invader LNAs, more labile) *versus* duplexes between L-modified ONs and cDNA (*i.e.*, probe-target duplexes, more stable) led us to stipulate that recognition of complementary dsDNA targets using Invader LNAs would be thermodynamically feasible and proceed *via* a double-duplex invasion process, provided that recognition kinetics are favorable.

**Fig. 8:**
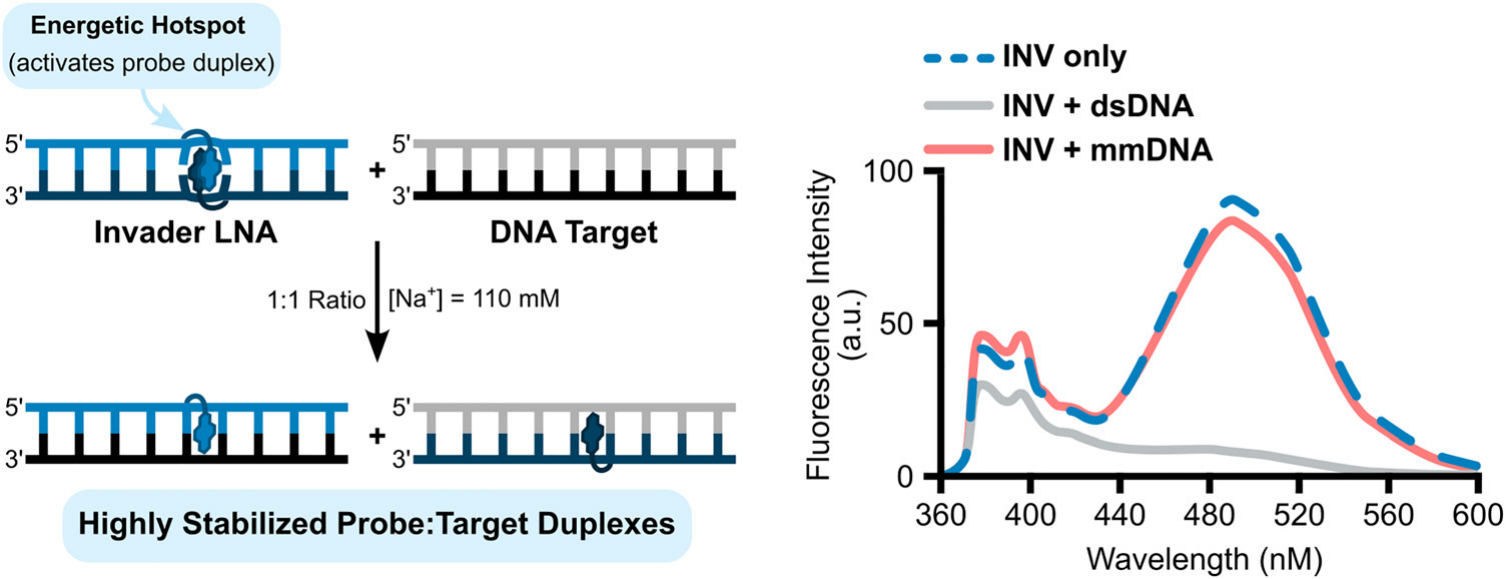
Illustrations of Invader LNA-mediated recognition of dsDNA and steady-state fluorescence emission spectra of an Invader LNA (INV) probe incubated with a complementary dsDNA (INV + dsDNA) or doubly-mismatched DNA (INV + mmDNA). Figure adapted from ref. 54 with permission from the Royal Society of Chemistry.

In a preliminary proof-of-concept experiment, a pre-annealed 9-mer Invader LNA featuring a single +1 interstrand zipper of L monomers was incubated with an equimolar quantity or twofold excess of a pre-annealed, unmodified DNA duplex (mixed-sequence, complementary to the probe; Fig. 8).^54^ A rapid (<1 min) disappearance of the pyrene excimer signal was observed, indicating that the Invader LNA probe had dissociated to form two corresponding probe-target duplexes (Fig. 8). Importantly, the experiments were performed in *T*_m_ buffer (formulated to mimic physiologic salinity: [Na^+^] = 110 mM) at temperatures significantly below the *T*_m_s of the Invader LNA probe, the dsDNA target, or any of the probe-target duplexes that would form upon successful recognition, indicating that the recognition process might proceed *via* a sequential displacement (strand invasion) process rather than a simple dissociative (strand exchange) process.^70^ Conversely, incubation of the Invader LNA probe with an equimolar quantity of a 9-mer DNA duplex differing in sequence at one or two positions relative to the probe (referred to as singly- or doubly-mismatched dsDNA targets despite being fully base-paired), resulted in much smaller decreases in excimer emission (Fig. 8).

Following our initial proof-of-concept study with Invader LNA probes,^54^ dsDNA-recognition using a closely related approach based on so-called intercalating nucleic acids (INAs) was reported.^71^ INAs are double-stranded ON probes with opposing bulges of non-nucleotidic 1-*O*-(1-pyrenylmethyl)glycerol monomers (see Fig. 2, bottom right corner). Akin to Invader LNA probes, this design results in a partially unwound and destabilized probe duplex^65^ whereas individual probe strands display high cDNA affinity, providing the driving force for dsDNA-recognition.

**Key point:** Invader LNA probes enable specific recognition of short, complementary mixed-sequence dsDNA at physiologically relevant salt concentrations.

### Invader LNAs with one or two energetic hotspots – preliminary insights into probe optimization

To further assess the viability of this approach for recognition of linear, mixed-sequence dsDNA targets, seven 13-mer Invader LNAs featuring one or two +1 zippers of L monomers were synthesized.^63^ Importantly, similar results were observed, validating the initial findings. Thus, duplexes between individual probe strands and cDNA were found to be significantly stabilized (Δ*T*_m_ per modification (Δ*T*_m_/mod) values between +7.0 °C and +11.8 °C). ONs with two L monomers as next-nearest neighbors displayed slightly less-than-additive increases in Δ*T*_m_ values, presumably as the local intercalator density approached the NNEP limit (*i.e.*, two intercalators per four base pairs). Conversely, more-than-additive stabilization was observed when two L monomers were separated further apart. The duplex stabilization was found to be due to less unfavorable changes in entropy compared to formation of the corresponding unmodified DNA duplex, likely reflecting the conformationally restricted nature of the bicyclic 2′-amino-α-l-LNA scaffold, which forces the attached pyrene moiety into the duplex core. Duplexes between individual probe strands and cRNA were found to be far less stabilized (Δ*T*_m_/mod between +0.5 °C and +6.3 °C), consistent with the preliminary study.

Invader LNAs featuring a single energetic hotspot were found to be quite labile (Δ*T*_m_ = −1.5 °C to +3.5 °C), as in the preliminary study. The lability is due to a less favorable change in enthalpy, consistent with localized intercalator-mediated perturbation of base-pairing and violation of the NNEP. Invader LNAs with two hotspots were found to be quite stable, although *T*_m_ and Δ*G*^293^ values (*i.e.*, the change in Gibbs free energy at 293 K upon duplex formation) provided conflicting indications (Δ*T*_m_ = +10.5 °C to +15.5 °C and ΔΔ*G*^293^ = 1–2 kJ mol^−1^, relative to the corresponding unmodified dsDNA).^63^

The free energy for recognition of isosequential dsDNA targets at 293 K by the different Invader LNA probes was estimated as follows: Δ*G*^293^_rec_ = Δ*G*^293^ (5′-ON:cDNA) + Δ*G*^293^ (3′-ON:cDNA) − Δ*G*^293^ (5′-ON:3′-ON) − Δ*G*^293^ (dsDNA), where 5′-ON:3′-ON denotes an Invader LNA. Invader LNAs featuring a single hotspot displayed Δ*G*^293^_rec_ values between −26 kJ mol^−1^ and −19 kJ mol^−1^, while probes with two hotspots were more strongly activated for dsDNA-recognition (Δ*G*^293^_rec_ values between −50 kJ mol^−1^ and −34 kJ mol^−1^). Probes with two non-consecutive hotspots were found to display more favorable Δ*G*^293^_rec_ values than probes with two sequential hotspots. Similar conclusions were drawn from the *T*_m_-based equivalent of the Δ*G*_rec_ term, *i.e.*, thermal advantage (TA) calculated as TA = *T*_m_ (5′-ON:cDNA) + *T*_m_ (3′-ON:cDNA) − *T*_m_ (5′-ON:3′-ON) − *T*_m_ (dsDNA) (note TA = −DA).

The impact of buffer ionic strength on the driving force for dsDNA-recognition was also assessed; Invader LNAs with two hotspots exhibited increasingly favorable Δ*G*^293^_rec_ values with decreasing ionic strength, whereas Δ*G*^293^_rec_ values for single hotspot Invader LNAs remained essentially unaffected by changes in ionic strength. These trends arise because individual probe strands display additionally increased cDNA affinity relative to unmodified strands as ionic strength is decreased, whereas the relative stability of Invader probes only is minimally impacted. Consequently, recognition of dsDNA targets with densely modified Invader LNAs is expected to become more thermodynamically favorable in environments with low ionic strength, with potential important ramifications for recognition of chromosomal DNA.

**Fig. 9:**
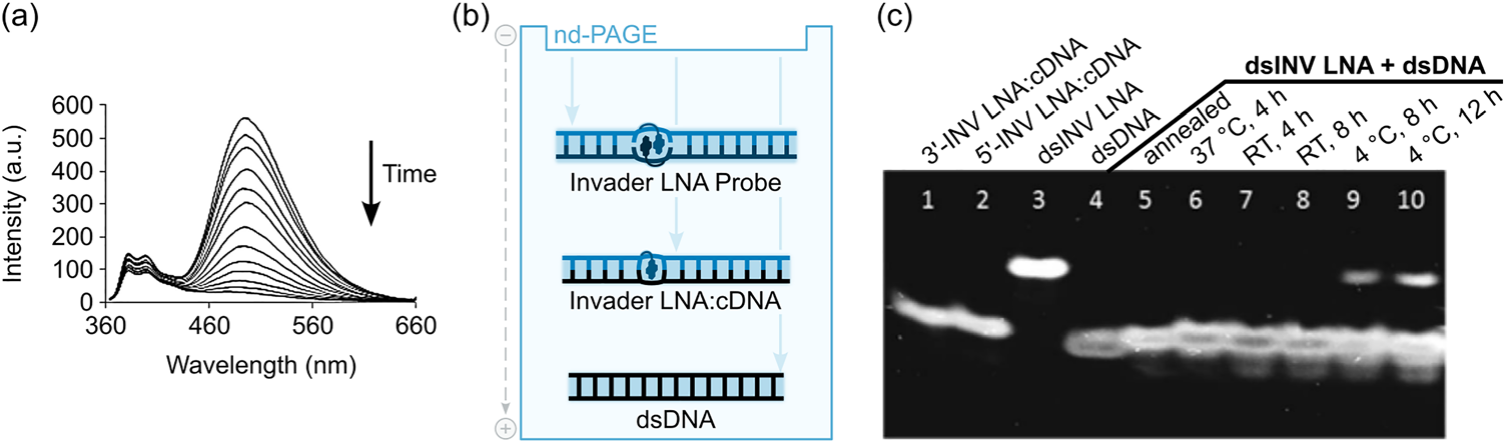
dsDNA-recognition properties of Invader LNA probes. (a) Time course of steady-state fluorescence spectra upon addition of a 13-mer pre-annealed Invader LNA to pre-annealed isosequential dsDNA target. (b) Depiction of the different mobilities of Invader LNA probes, probe-target duplexes between Invader LNA strands and cDNA, and dsDNA in non-denaturing polyacrylamide gels. (c) Representative electrophoretogram demonstrating dsDNA-recognition by Invader LNA probes. Figure reproduced from ref. 63 with permission from the Royal Society of Chemistry.

A subset of these 13-mer Invader LNAs were pre-annealed and then incubated with an equimolar quantity of the corresponding pre-annealed dsDNA targets at room temperature, *i.e.*, at an experimental temperature well below the *T*_m_s of the dsDNA target (37.5 °C), Invader LNAs (36–53 °C), and probe-target duplexes (44.5–61.0 °C) that would form upon successful recognition. The recognition kinetics were monitored by observing the decrease in pyrene excimer emission (Fig. 9a). The recognition process was 50% complete (*t*_50%_) within 29–48 min when experiments were performed in *T*_m_ buffer ([Na^+^] = 110 mM). Only minor differences were observed between the Invader LNAs despite their different Δ*G*^293^_rec_ values, suggesting that target recognition – in this instance – is not thermodynamically limited. Conversely, when a representative 13-mer single hotspot Invader LNA was incubated with dsDNA targets differing in sequence at one position, a slower and less pronounced decrease in the excimer signal was observed. This was consistent with the unfavorable thermodynamics for recognition of mismatched targets (Δ*G*^293^_rec_ values between −1 kJ mol^−1^ and +8 kJ mol^−1^) and suggested that Invader LNA-mediated recognition of mixed-sequence dsDNA targets is highly specific.

Invader LNA probes featuring two sequential hotspots were found to result in significantly faster dsDNA-recognition than single hotpot Invader LNAs in high salt ([Na^+^] = 710 mM) or KMST buffers (pH 7.2, 140 mM KCl, 10 mM MgCl_2_, 1 mM spermine and 40 mM Tris-Cl) (*t*_50%_ = 126/31 min and 158/19 min, for singly/doubly modified Invader LNAs in high salt and KMST buffers, respectively). These findings not only suggested that dsDNA-recognition is possible across a broad range of conditions but also hinted at preferred probe designs.

To validate the results from the fluorescence-based assay, electrophoretic mobility shift assays (EMSAs) entailing non-denaturing polyacrylamide gel electrophoresis (nd-PAGE) were conducted. The EMSAs rely on differences in the electrophoretic mobilities of Invader LNAs (lowest mobility; most elongated duplex due to double intercalation), isosequential dsDNA targets (highest mobility; most compact duplex), and probe-target duplexes (intermediate mobility and elongation) (Fig. 9b). A representative 13-mer single hotspot Invader LNA was incubated with an equimolar amount of the corresponding isosequential dsDNA target (4 h, at either 37 °C or room temperature, in a pH 7.2 HEPES buffer (70 mM HEPES, 15 mM MgCl_2_, 15% sucrose, 0.15% spermine, 300 mM NaCl)), leading to the formation of probe-target duplexes as evidenced by a single band with the same mobility as the pre-annealed probe-target reference duplexes (compare lanes 6 and 7 with lanes 1, 2, and 5, Fig. 9c). No band corresponding to the Invader LNA (lane 3, Fig. 9c) was present, indicating these conditions facilitated complete recognition of the dsDNA target. Recognition proceeded, but remained incomplete, following incubation at 4 °C for 8–12 hours, as indicated by the presence of a faint band corresponding to the Invader LNA (compare lanes 9 and 10 with lane 3, Fig. 9c).

**Key point:** 13-Mer Invader LNA probes featuring hotspots of L monomers allow for fast and specific recognition of complementary dsDNA targets across different buffers – including buffers mimicking physiological conditions and high salt buffers. The difference in stability of the probe-target duplexes relative to the Invader LNAs and dsDNA targets renders recognition thermodynamically favorable. Invader LNAs with two hotspots display more favorable recognition thermodynamics and faster dsDNA-recognition kinetics than single hotspot Invader LNAs. The recognition process can be followed by monitoring the disappearance of the probe's pyrene excimer signal or by comparing relative mobilities in EMSAs.

## Second-generation Invader probes

### Identifying and synthesizing more readily available Invader LNA mimics

The initial studies highlighted the potential of Invader LNAs for sequence-unrestricted recognition of dsDNA targets. However, a comprehensive evaluation of these probes was not feasible due to the complex synthesis and limited supply of the necessary N2′-pyrene-functionalized 2′-amino-α-l-LNA phosphoramidites (Schemes 1 and 3). Consequently, our attention shifted to identifying and obtaining more readily available structural and functional mimics of N2′-pyrene-functionalized 2′-amino-α-l-LNA monomers. A survey of the existing literature at the time (approximately 2005–2010) suggested O2′-pyrene-functionalized RNA and N2′-pyrene-functionalized 2′-*N*-methyl-2′-amino-DNA monomers as potential candidates. ONs modified with 2′-*O*-(pyren-1-yl)methyluridine monomer X^57^ or 2′-*N*-methyl-2′-*N*-(pyren-1-yl)methyl-2′-aminodeoxyuridine monomer Y^58^ (Fig. 3) had been shown to exhibit high cDNA affinity. Moreover, the pyrene moiety of monomer X had been shown to intercalate in a 3′-directed manner upon hybridization with cDNA,^72^ in a similar manner as proposed for monomer L (Fig. 5). Although unknown to us at the time, the high cDNA affinity bestowed by the pyrene moieties of monomers L, X, and Y, is likely linked to their sugar rings adopting *South*-type conformations. The next task then involved developing convenient synthetic routes to the corresponding phosphoramidites.

**Scheme 4:**
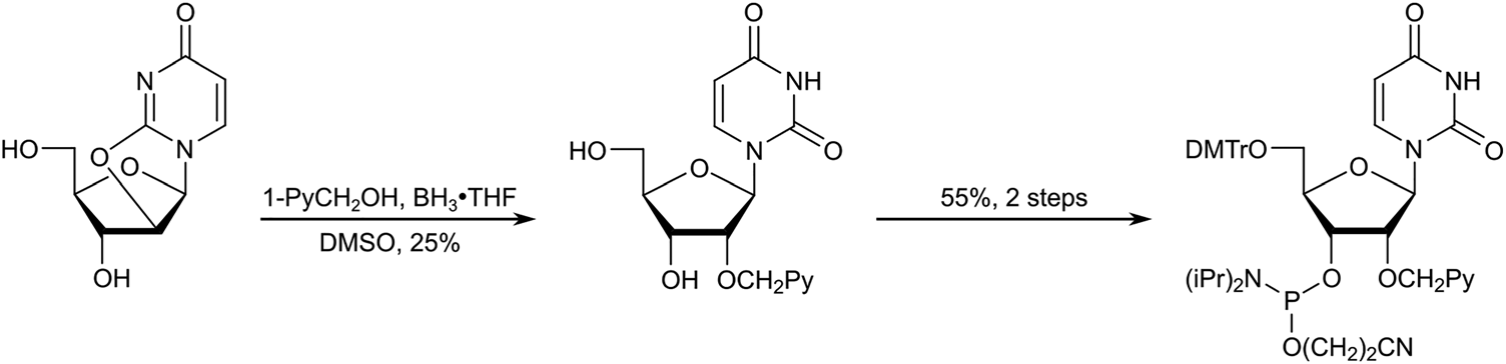
Outline of synthetic route to phosphoramidite of monomer X.

Yamana and co-workers reported a synthetic route to the corresponding phosphoramidite of monomer X.^73^ The approach entailed (i) O3′,O5′-ditritylation of uridine, (ii) installation of the pyrene moiety at the O2′-position *via* Williamson's ether synthesis, (iii) detritylation, (iv) O5′-dimethoxytritylation, and (v) O3′-phosphitylation. In our hands, the O3′,O5′-ditritylation only proceeded with modest regioselectivity and yield, and the protecting group manipulations following the O2′-functionalization were cumbersome. We therefore set out to develop an alternative route (Scheme 4).^74^ Specifically, trialkyl borate-mediated opening^75^ of O2,O2′-anhydrouridine using 1-pyrenemethanol in anhydrous DMSO afforded the O2′-pyrene-functionalized key intermediate in modest but consistent yields despite challenging column purifications. Subsequent O5′-dimethoxytritylation and O3′-phosphitylation, as reported by Yamana, afforded the target phosphoramidite in an overall yield of ∼12% over five steps from uridine, a modest improvement over the original route. Other intercalators were subsequently installed in an equivalent manner.^76–78^

**Scheme 5:**
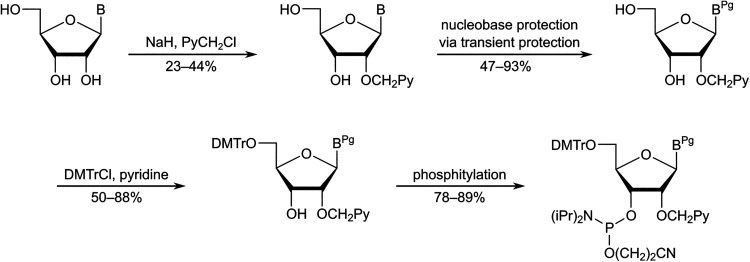
Outline of synthetic route to the phosphoramidites of the protected adenine, cytosine and guanine analogues of monomer X. Pg = protecting group.

The protected adenine, cytosine, and guanine analogues were synthesized in a different manner as reported by Yamana and us (Scheme 5).^79,80^ The free nucleosides were first O2′-pyrenylmethylated in modest regioselectivity and yield using Williamson's approach, followed by *N*-acylation of the exocyclic amines *via* transient protection protocols, O5′-dimethoxytritylation, and O3′-phosphitylation. This reaction sequence afforded the necessary phosphoramidites in overall yields ranging from 9% to 12% over four steps from the free nucleosides.

The Wengel group reported the synthesis of the corresponding phosphoramidite of monomer Y from 5′-*O*-dimethoxytrityl-2′-aminouridine in approximately 7% overall yield over five steps.^58^ The route entailed N2′-pyrene-functionalization *via* reductive amination, O5′-detritylation, N2′-methylation under acidic conditions, O5′-dimethoxytritylation, and O3′-phophitylation.

**Scheme 6:**
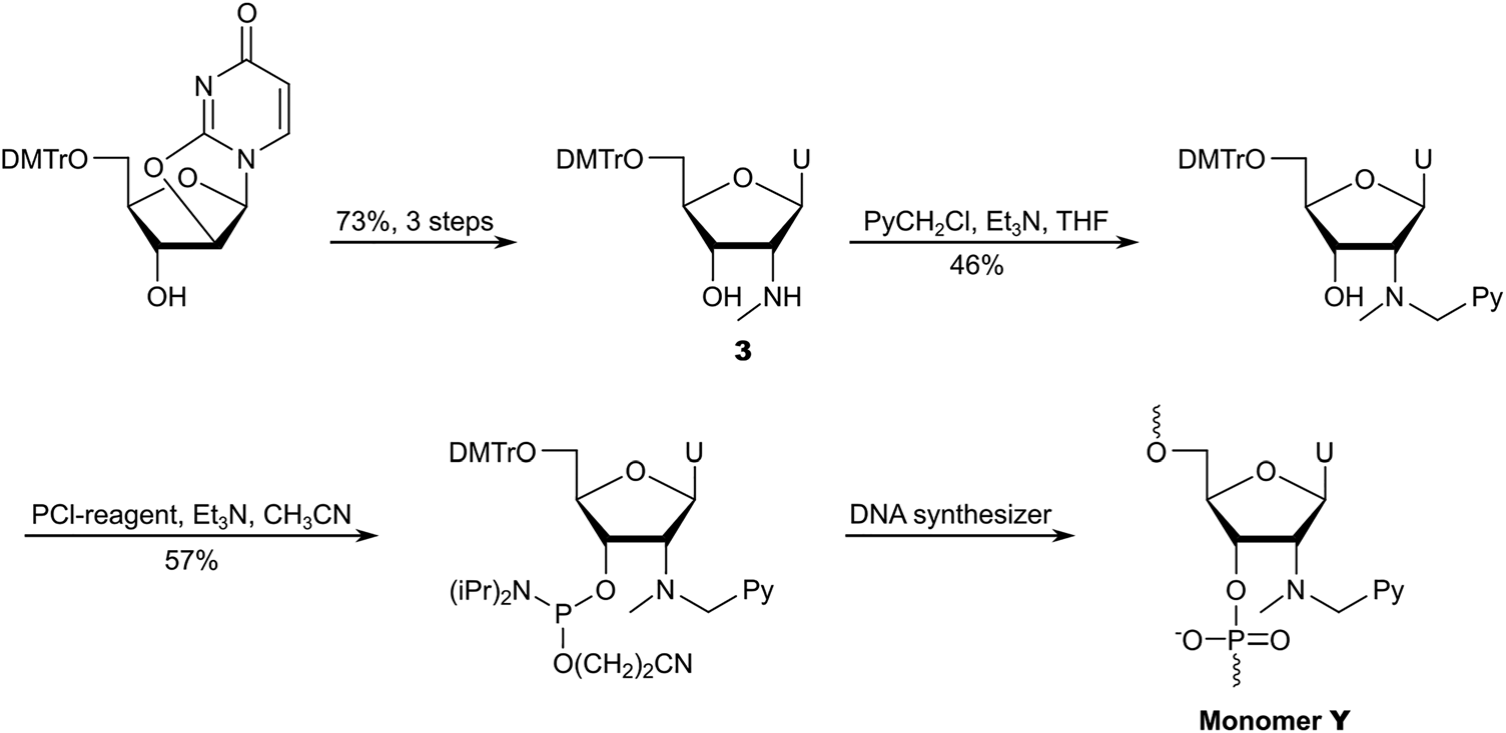
Outline of our initial synthetic route to the phosphoramidite of monomer Y.

To reduce the number of protection/deprotection steps, we decided to use nucleoside 3^74^ as a starting material instead, which is obtained from 5′-*O*-dimethoxytrityl-2,2′-anhydrouridine in ∼73% yield over three steps (Scheme 6).^81^ Direct N2′-alkylation of 3 using 1-(chloromethyl)pyrene afforded the desired product in moderate yield, whereas reductive amination proved unsuccessful due to the formation of cyclic N2′,O3′-hemiaminal ethers.^74^ Subsequent O3′-phosphitylation provided the target phosphoramidite in 10% overall yield from uridine over seven steps.

**Scheme 7:**
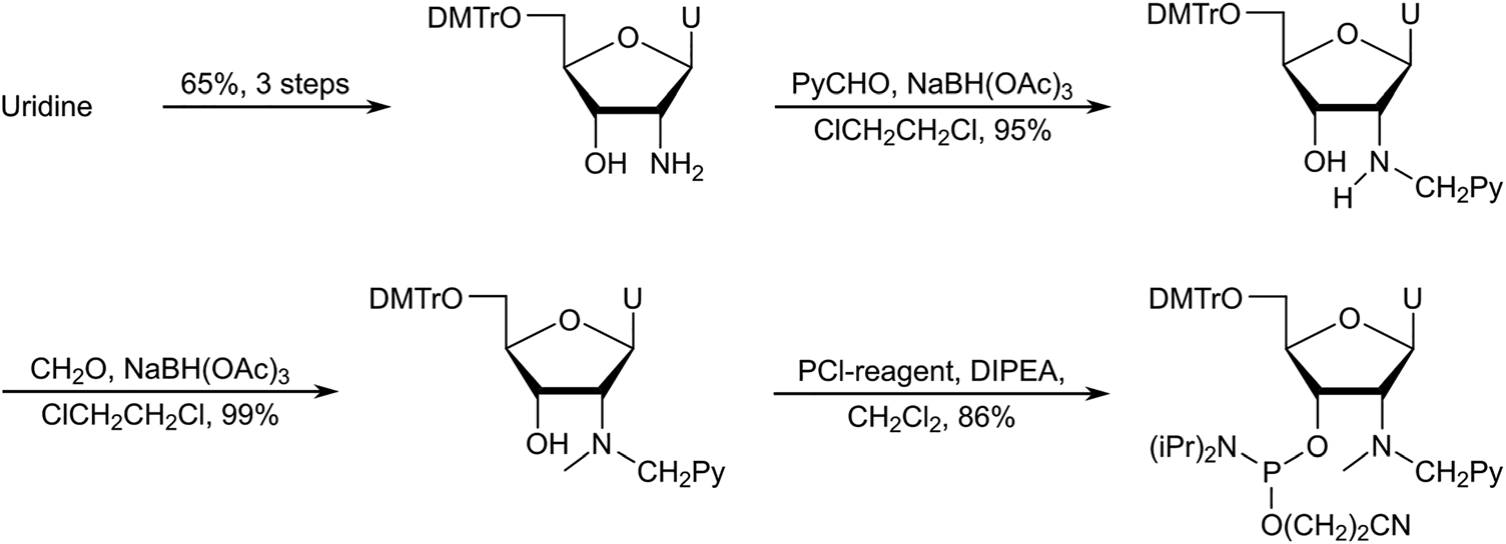
Improved synthetic route to the phosphoramidite of monomer Y.

After establishing monomer Y as a valuable building block for second-generation Invader probes (*vide infra*), we developed an improved route to the corresponding phosphoramidite (Scheme 7).^82^ Using 5′-*O*-dimethoxytrityl-2′-amino-2′-deoxyuridine as a starting material^83^ – which was obtained from uridine in three steps – we performed consecutive reductive aminations, first introducing the large N2′-substituent, then the N2′-methyl group. Improved O3′-phosphitylation afforded the phosphoramidite of monomer Y in ∼52% yield from uridine over six steps,^82^ representing a significant improvement over the initial route. This route also allowed for the introduction of other intercalators at the N2′-position.^82^

**Key point:** 2′-*O*-(Pyren-1-yl)methylribonucleoside and 2′-amino-2′-deoxy-2′-*N*-methyl-2′-*N*-(pyren-1-yl)methyluridine phosphoramidites can be obtained in far fewer steps and significantly higher overall yield than N2′-pyrene-functionalized 2′-amino-α-l-LNA phosphoramidites.

### Characterizing ONs modified with X or Y monomers

The impact of the X and Y monomers on duplex stability was examined alongside the α-l-LNA-T (monomer O), unfunctionalized 2′-amino-α-l-LNA-T (monomer N), and 2′-*N*-(pyren-1-yl)methyl-2′-amino-α-l-LNA-T (monomer L) monomers.^62,74^ Importantly, duplexes between X- or Y-modified ONs and cDNA were found to be only slightly less stable than the corresponding L-modified duplexes, but more stable than the corresponding O- and N-modified duplexes (Table 1). The stability trends of DNA duplexes with different interstrand zipper arrangements of monomers X and Y were found to closely mirror those of corresponding L-modified duplexes. Thus, DNA duplexes with −3, −1, and +4 interstrand zippers of X or Y monomers exhibit *T*_m_ values consistent with near-additive contributions from the two monomers. Duplexes with +2 zippers display *T*_m_s indicative of moderately destabilizing interference between the two monomers, while duplexes with +1 zippers are as labile and with as strongly destabilizing interference as the corresponding Invader LNAs (compare *T*_m_ and TA values, Table 1).

**Table 1:** Δ*T*_m_ and TA values for Invader probe duplexes featuring O, N, L, X, or Y monomers in +4 or +1 zippers, and Δ*T*_m_ values for duplexes between probe strands and cDNA

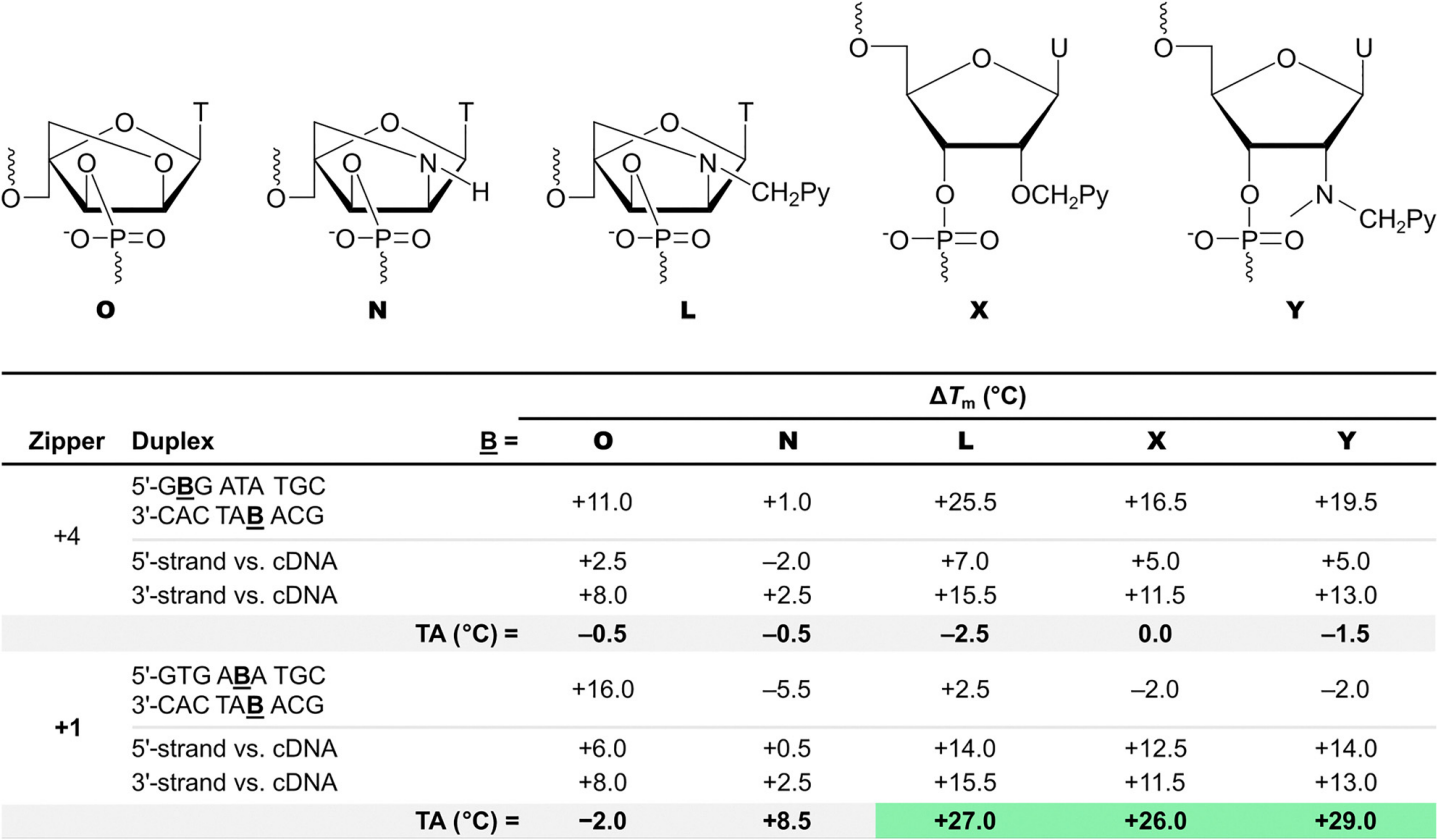

The similar thermal denaturation characteristics of the X-, Y-, and L-modified duplexes indicated to us that the pyrene moieties have similar binding modes, *i.e.*, stabilizing intercalation in duplexes between the pyrene-modified ONs and cDNA, and destabilizing interference and violation of the NNEP in DNA duplexes with +1 zippers. The following observations corroborated this further:

(i) X- and Y-modified ONs display similar selectivity for cDNA over cRNA as L-modified ONs^74^

(ii) The increased stability of duplexes between X-modified ONs and cDNA is also due to less unfavorable changes in entropy upon duplex formation^76^

(iii) X- and Y-modified ONs also display reduced discrimination of DNA strands with mismatched nucleotides positioned opposite the pyrene-functionalized monomer^74^

(iv) Hybridization of X- and Y-modified ONs with cDNA also results in bathochromic shifts of pyrene absorption maxima^74^

**Fig. 10:**
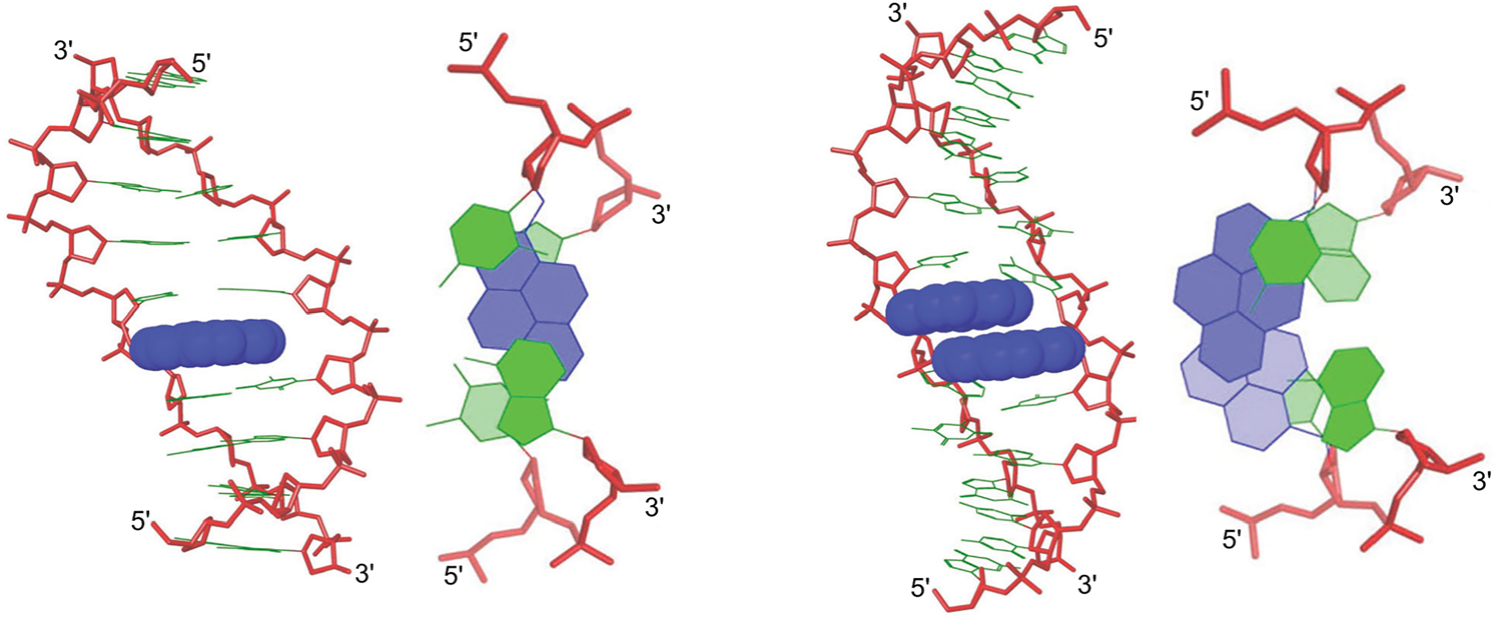
Side and top views of low energy structures for a duplex between an X-modified ON and cDNA (left) and a DNA duplex with a +1 zipper of X monomers (right). Reproduced from ref. 76 with permission from the Royal Society of Chemistry.

(v) Hybridization of X- or Y-modified ONs with cDNA leads to reduced fluorescence emission from the pyrene moieties,^74,76^ consistent with nucleobase-mediated quenching of emission from intercalating pyrenes^84,85^

(vi) The pyrene moiety of a duplex between an X-modified ON and cDNA remained intercalated throughout a 5 ns molecular dynamics simulation (Fig. 10, left),^76^ consistent with earlier NMR studies suggesting 3′-directed intercalation of the pyrene moiety^72^

(vii) The destabilization of duplexes with +1 interstrand zippers of X monomers is also due to less favorable changes in enthalpy^76^

(viii) DNA duplexes with +1 zippers of X monomers exhibit blue-shifted pyrene absorption maxima relative to DNA duplexes with other zippers of X monomers or duplexes between X-modified ONs and cDNA, indicative of disrupted stacking interactions between pyrenes and nucleobases^76^

(ix) Steady-state fluorescence emission spectra of DNA duplexes with +1 interstrand zippers of X or Y monomers also display excimer signals at *λ*_em_ ∼ 490 nm, albeit less distinctly than the corresponding L-based Invader LNAs.^62^ Interestingly, DNA duplexes with +2 interstrand zipper arrangements of X monomers, display a more prominent excimer signal, presumably due to pyrene–pyrene stacking in the major groove,^62^ in a similar manner as observed for DNA duplexes with +2 zipper arrangements of pyrene-functionalized ara-uridine monomers.^86–88^

(x) Molecular dynamics simulations suggest that DNA duplexes with +1 zippers of X monomers are distorted. Similarly to the observations with Invader LNAs, two possible binding modes appear feasible, *i.e.*, one where both pyrenes intercalate into the duplex core (Fig. 10, right) and another where one pyrene intercalates while the other points into the major groove. The observed excimer emission suggests that forced intercalation of both pyrene moieties occurs (first mode), but the weak intensity of this emission suggests that the second mode is also adopted. Both binding modes can explain the labile nature of the double-stranded probes, as base pairing is distorted near the pyrene zipper in either duplex structure. Moreover, the presence of a large hydrophobic pyrene in the major grove (as in the second mode) likely perturbs the hydration spine of the duplex.^76^

**Key point:** Duplexes modified with 2′-*O*-(pyren-1-yl)methyl-uridine monomer X or 2′-*N*-methyl-2′-*N*-(pyren-1-yl)methyl-2′-aminodeoxyuridine monomer Y display very similar thermal denaturation characteristics as corresponding duplexes modified with 2′-*N*-(pyren-1-yl)methyl-2′-amino-α-l-LNA-T monomer L. Biophysical characterization and molecular modeling simulations suggest that the pyrene moieties of the three monomers adopt similar binding modes.

### Preliminary dsDNA-recognition experiments using second-generation Invader probes

**Fig. 11:**
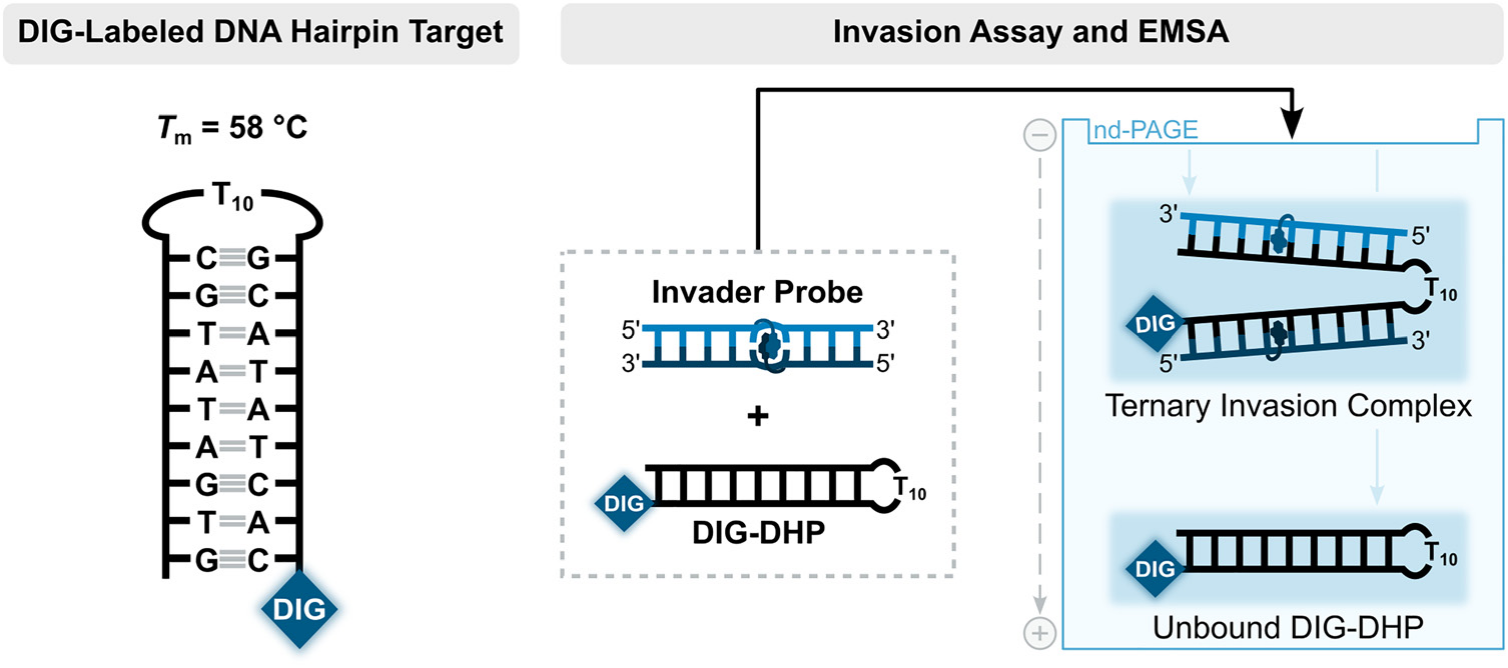
Illustration of a model digoxigenin (DIG)-labeled DNA hairpin (DHP) target and the invasion assay used to assess dsDNA-recognition by Invader probes.

Having confirmed that the X and Y monomers display similar thermal denaturation properties and pyrene binding modes as monomer L, we proceeded to evaluate the dsDNA-targeting properties of Invader probes constructed using these monomers. Towards this end, a 3′-digoxigenin (DIG)-labeled DNA hairpin (DHP) – featuring a 9-mer double-stranded stem that is complementary to the Invader probes and linked at one end through a decameric thymidine (T_10_) loop – was used as a model target (Fig. 11).^62^ The unimolecular and high-melting nature of the DNA hairpin (*T*_m_ = 58 °C) ensures that both target strands are present in identical amounts and less likely to fray than a corresponding linear dsDNA target. Invasion of the double-stranded stem region is expected to result in the formation of a ternary invasion complex (IC) that has a lower mobility than the unbound DNA hairpin when incubation mixtures are resolved by nd-PAGE.

**Fig. 12:**
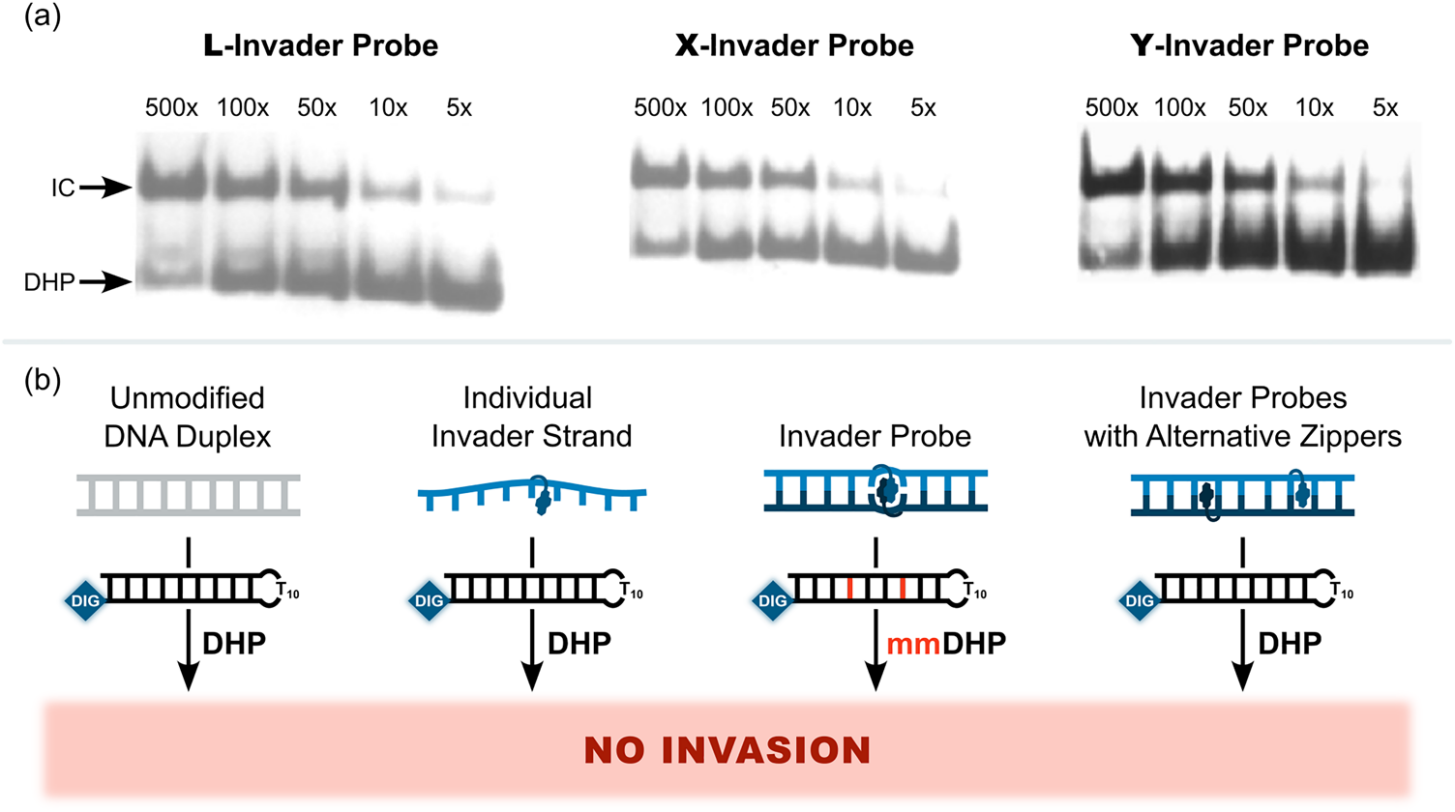
(a) Representative electrophoretograms from recognition experiments in which a DIG-DHP was incubated with varying probe excesses of L-, X-, or Y-modified probes. (b) Illustration depicting assay conditions that do not result in invasion of the DHP target. “IC” = invasion complex. “mmDHP” is a fully base-paired DNA hairpin that differs in sequence at two positions relative to the Invader probe. Data shown in panel a is from ref. 62, with permission from the American Chemical Society, copyright 2013.

Importantly, L-, X-, and Y-based Invader probes were found to display near-identical recognition characteristics. Thus, 45–48% recognition of the DNA hairpin was observed when it was incubated with a 100-fold molar probe excess at room temperature (Fig. 12a).^62^ However, an Invader probe constructed using the adenine analogue of monomer X, resulted in more efficient recognition of the hairpin target than the corresponding probe based on the adenine analogue of monomer L (41% *vs.* 17% recognition, 100-fold molar excess), possibly as the former is more energetically activated for dsDNA-recognition (TA = 20.5 °C *vs.* 18.5 °C, respectively) due to a slightly higher cDNA affinity.^62^

None of the following control experiments resulted in significant target recognition: (i) incubation of the DNA hairpin target with a 500-fold molar excess of a corresponding unmodified 9-mer DNA duplex, (ii) incubation of the DNA hairpin target with a 100-fold molar excess of individual Invader strands, or (iii) incubation of fully base-paired DNA hairpins differing in sequence at one or two positions relative to Invader probes used at 100-fold excess (Fig. 12b).^62^ Subsequent studies revealed that double-stranded probes with +5 or −3 interstrand zippers of X monomers also fail to recognize the corresponding DNA hairpin target – at conditions in which corresponding probes with a +1 zipper resulted in substantial recognition.^89^ These results demonstrated that (i) the presence of energetic hotspots is indispensable for activating Invader probes for mixed-sequence dsDNA-recognition, (ii) both strands of an Invader probe are needed for efficient dsDNA-recognition, and (iii) recognition of dsDNA targets using Invader probes proceeds with excellent binding specificity.

**Key point:** 2′-*O*-(Pyren-1-yl)methyl-uridine monomer X and 2′-*N*-methyl-2′-*N*-(pyren-1-yl)methyl-2′-aminodeoxyuridine monomer Y are suitable building blocks for the construction of Invader probes, and display near-identical dsDNA-recognition characteristics as Invader probes based on 2′-*N*-(pyren-1-yl)methyl-2′-amino-α-l-LNA-T monomer L, including excellent binding specificity. Identification of these second-generation building blocks opened the possibility for optimization of probe designs and the use of Invader probes for exploratory diagnostic applications.

## Establishing design rules for second-generation Invader probes

### The nucleobase composition of the energetic hotspot impacts the stability and dsDNA-recognition efficiency of Invader probes

With more easily accessible second-generation Invader monomers available, we were able to systematically study the impact that the nucleobase moieties of these monomers have on the dsDNA-recognition efficiency of Invader probes. Towards this end, ten Invader probes were designed, differing only in the composition of a central energetic hotspot constructed using 2′-*O*-(pyren-1-yl)methyl-RNA monomers (Table 2). The inherent symmetry of the energetic hotspots, along with the utilized sequence context – *i.e.*, hotspots flanked by A:T pairs on either side – allowed us to study the sixteen possible hotspot compositions using only ten probes.

**Table 2:** Δ*T*_m_, TA, ΔΔ*G*^293^, and Δ*G*^293^_rec_ values for Invader probes and duplexes between individual Invader strands and cDNA with varying hotspot compositions^a^

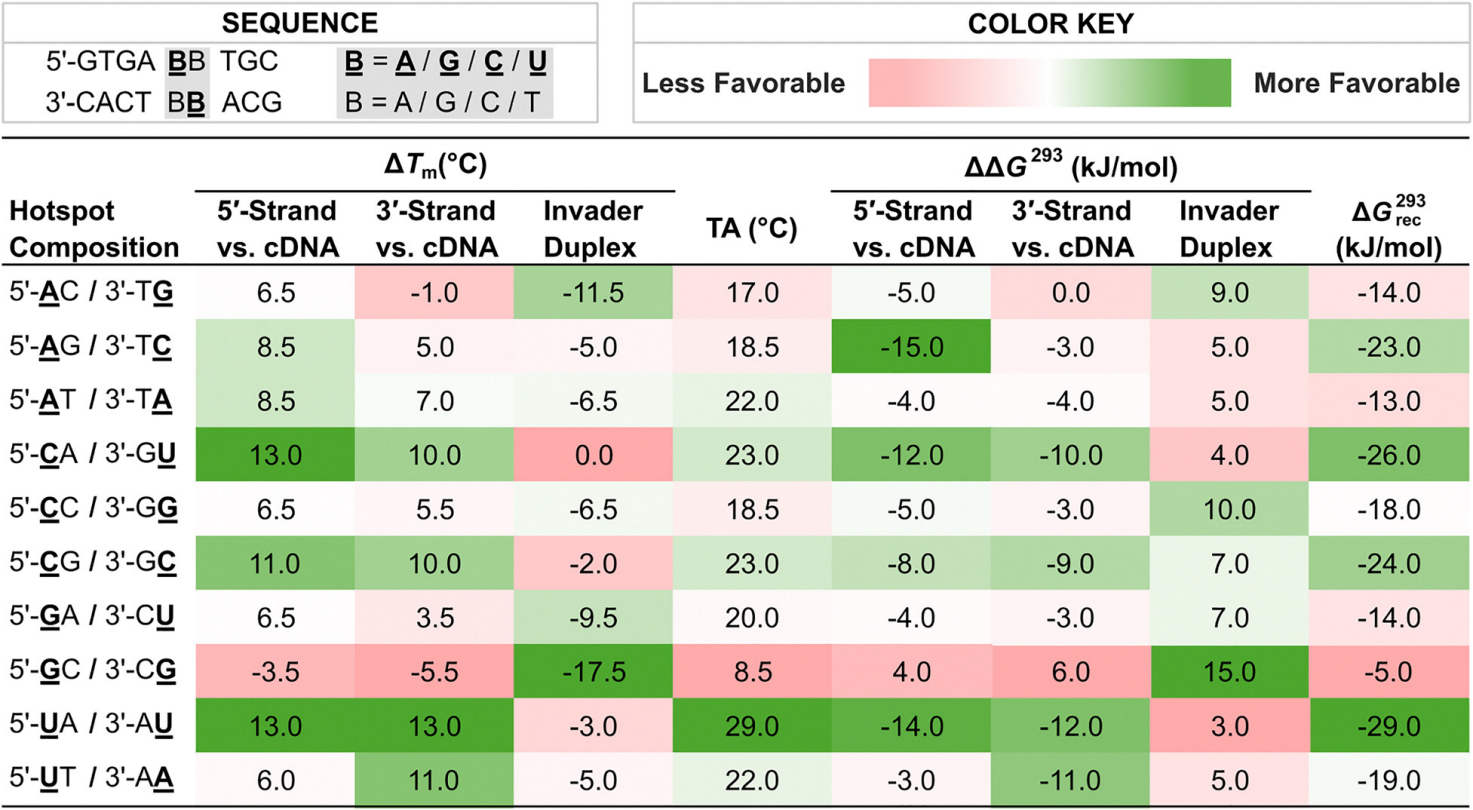

a A̲, G̲, C̲, and U̲ are 2′-*O*-(pyren-1-yl)methyl-RNA monomers with adenin-9-yl, guanin-9-yl, cytosin-1-yl, and uracil-1-yl nucleobases, respectively. Hotspot compositions correspond to the sequence 5′-GTGA-**B̲**B-TGC/3′-CACT-B**B̲**-ACG where B̲ and B are the indicated Invader and DNA monomers, respectively.

Each of these Invader probes was found to display reduced stability relative to the corresponding unmodified DNA duplexes with Δ*T*_m_ values ranging between −17.5 °C and 0.0 °C, and ΔΔ*G*_293_ values between +3 and +15 kJ mol^−1^, Table 2).^79^ Probes with 2′-*O*-(pyren-1-yl)methyl-RNA-G monomers were particularly labile. Thus, +1 interstrand zippers of 2′-*O*-(pyren-1-yl)methyl-RNA monomers appear to disrupt stacking and nearby base pairs, irrespective of the hotspot's nucleobase composition. Further support for this was provided by the following characteristics that were observed for each Invader probe: (i) presence of pyrene excimer emission in steady-state fluorescence emission spectra, (ii) broad denaturation profiles indicating a distorted duplex, and (iii) blue-shifted pyrene absorption maxima (Δ*λ* = 1–9 nm) relative to duplexes between individual probe strands and cDNA.^79^

Duplexes between individual Invader strands modified with 2′-*O*-(pyren-1-yl)methyl-RNA-A/C/U monomers and cDNA were found to be strongly stabilized compared to the corresponding unmodified DNA duplexes (Δ*T*_m_ values between +3.5 °C and +13.0 °C, and ΔΔ*G*^293^ values between −3 kJ mol^−1^ and −15 kJ mol^−1^, Table 2).^79^ In contrast, probe-target duplexes are less stable if modified with 2′-*O*-(pyren-1-yl)methyl-RNA-G monomers (Δ*T*_m_ values between −5.5 °C and +6.5 °C; ΔΔ*G*^293^ values between +6 kJ mol^−1^ and −4 kJ mol^−1^). Probe-target duplex stability is also impacted by the nature of the 3′-flanking nucleotide relative to the 2′-*O*-(pyren-1-yl)methyl-RNA monomer, in a similar manner as observed with 2′-*N*-(pyren-1-yl)methyl-2′-amino-α-l-LNA monomers.^54^ Thus, greater stabilization is observed when 2′-*O*-(pyren-1-yl)methyl-RNA monomers are flanked by a purine on the 3′-side, consistent with 3′-directed pyrene intercalation.

As a result of the above factors, each of these Invader probes was found to exhibit favorable thermodynamics for the recognition of complementary dsDNA targets, albeit to varying degrees (Δ*G*^293^_rec_ values between −29 and −5 kJ mol^−1^, Table 2).^79^ Invader probes with energetic hotspots composed exclusively of 2′-*O*-(pyren-1-yl)methyl-RNA pyrimidine monomers display the most prominent driving force for dsDNA-recognition (Δ*G*^293^_rec_ values between −29 and −24 kJ mol^−1^), which reflects the greater probe-target duplex stability when 2′-*O*-(pyren-1-yl)methyl-RNA monomers are flanked by 3′-purines.^79^ In contrast, Invader probes with hotspots comprised exclusively of 2′-*O*-(pyren-1-yl)methyl-RNA-purine monomers – meaning each monomer is flanked by a 3′-pyrimidine − display the least favorable thermodynamics for dsDNA-recognition (Δ*G*^293^_rec_ values between −14 and −5 kJ mol^−1^).^79^ Invader probes with mixed pyrimidine/purine hotspots, fall between these two extremes (Δ*G*^293^_rec_ values between −23 and −14 kJ mol^−1^).^79^

Four 9-mer Invader probes, each featuring a central hotspot comprised of 2′-*O*-(pyren-1-yl)methyl-RNA U̲/U̲, C̲/C̲, U̲/A̲, and G̲/G̲ monomers (selected for their wide range of Δ*G*^293^_rec_ values and absolute *T*_m_ values), were evaluated using the DNA hairpin invasion assay discussed previously (Fig. 11). Incubation of these Invader probes with the respective DNA hairpin targets at room temperature resulted in the formation of invasion complexes, in which the extent of invasion was consistent with the Δ*G*^293^_rec_ values of the probes. Thus, the Invader probe with the most favorable Δ*G*^293^_rec_ value (U̲/U̲) resulted in ∼80% recognition of the hairpin target when used at a 100-fold excess, while the probe with the least favorable Δ*G*^293^_rec_ value (G̲/G̲) resulted in minimal target recognition.

**Fig. 13:**
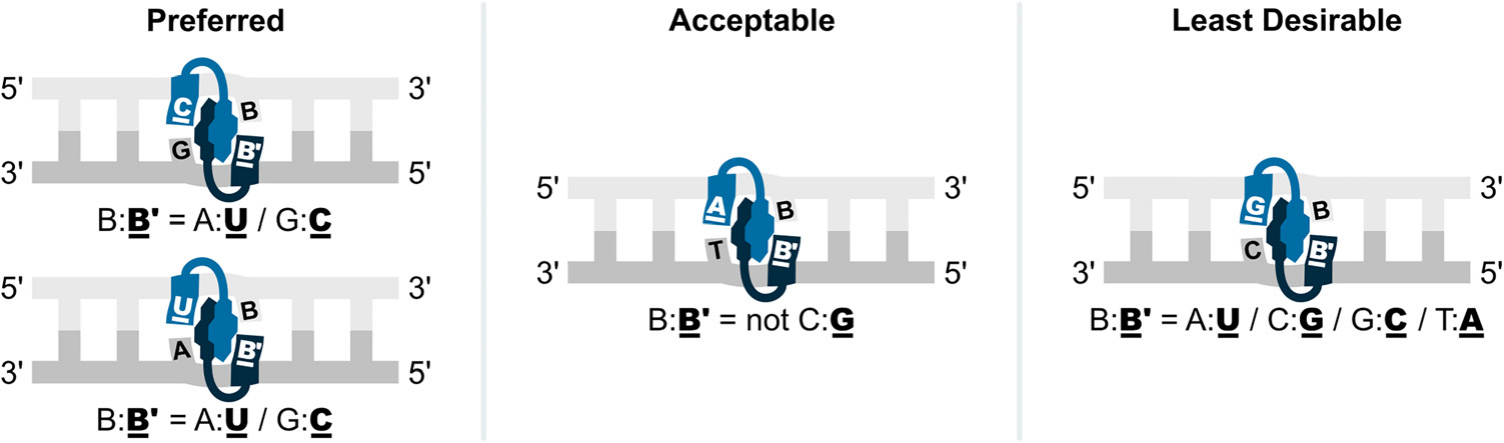
Illustration of preferred, acceptable, and least desirable Invader hotspot compositions. B′_ and B are an Invader monomer and the complementary DNA monomer.

Thus, these results suggested to us that the preferred hotspot design entails the use of pyrene-functionalized pyrimidine monomers (targeting 5′-CA/CG/TA/TG-3′ steps), although the corresponding adenine monomers are acceptable (targeting 5′-AA/AG/AT/CT/TT-3′ steps); the use of guanine monomers is less desirable (avoid targeting 5′-AC/CC/GA/GC/GG/GT/TC steps) (Fig. 13).

The design principles inferred from the 9-mer study were successfully applied to longer and more densely modified Invader probes. Four 14-mer Invader probes, each featuring three distributed energetic hotspots, were designed to yield probes with different dsDNA-recognition potential. Thus, one of these Invader probes featured energetic hotpots solely composed of 2′-*O*-(pyren-1-yl)methyl-RNA pyrimidine monomers (efficient target recognition expected), while a different Invader probe only featured energetic hotspots composed of 2′-*O*-(pyren-1-yl)methyl-RNA purine monomers (least target recognition expected). Dose-dependent recognition of the corresponding DNA hairpin target (GC content ∼43%) was observed at room temperature with each of these Invader probes, with trends following expectations. Interestingly, even the Invader probe exclusively featuring hotspots composed of 2′-*O*-(pyren-1-yl)methyl-RNA-purine monomers, resulted in some target recognition, indicating that a favorable probe architecture (*i.e.*, densely modified probes, *vide infra*), can overcome suboptimal hotspot selection.

**Key point:** Invader probes with energetic hotspots composed of 2′-*O*-(pyren-1-yl)methyl-RNA pyrimidine monomers display the most prominent dsDNA-recognition potential due to the high stability of the resulting probe-target duplexes where purines flank the monomers on the 3′-side. Conversely, 2′-*O*-(pyren-1-yl)methyl-RNA-G monomers are best avoided as individual probe strands display low cDNA affinity when cytidines flank the monomers on the 3′-side.

### Extensively modified second-generation Invader probes display improved dsDNA-recognition characteristics

Invader probes with different modification patterns (*i.e.*, variable number of, location of, and distance between energetic hotspots) were constructed using second-generation monomers in a preferred, TA-alternating sequence context and studied to understand how probe designs impact dsDNA-recognition.^90^ Twenty 13-mer Invader probes, modified with X or Y monomers were synthesized toward this end (Table 3).

**Table 3:** TA and Δ*G*^293^_rec_ values for different X- and Y-modified probe designs

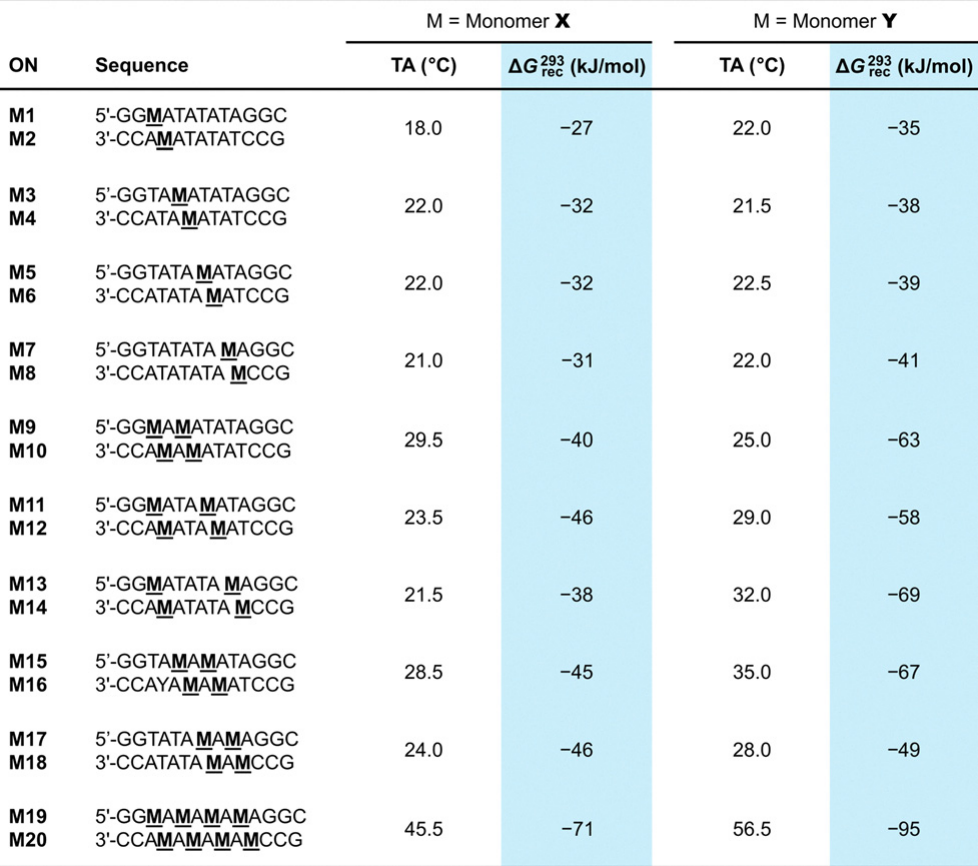

As in the preliminary studies, singly modified ONs formed greatly stabilized duplexes with cDNA relative to unmodified ONs, with Y-modified ONs consistently forming slightly more stable duplexes (Δ*T*_m_ = 7.0–11.0 °C *vs.* 8.0–13.5 °C for X- and Y-modified duplexes, respectively).^90^ Incorporation of a second pyrene-functionalized monomer resulted in further stabilization (Δ*T*_m_ = 14.0–21.5 °C) although the observed *T*_m_ increases were slightly less than additive, suggesting that intercalation of a first pyrene moiety, to a minor degree, impacts the stabilizing properties of a second pyrene moiety. This trend becomes progressively more pronouced with increasing modification levels.

Invader probes with a single energetic hotspot were found to display similar *T*_m_s as the corresponding unmodified DNA duplexes (Δ*T*_m_ = −4.0 to +3.0 °C),^90^ while probes with two or four hotspots were moderately stabilized (Δ*T*_m_ = +2.0 to +12.5 °C), suggesting that the presence of a first energetic hotspot reduces the destabilizing characteristics of subsequent hotspots. Lower *T*_m_s were generally observed for Y-modified probes and probes with consecutive energetic hotspots, mirroring observations for Invader probes modified with the original 2′-*N*-(pyren-1-yl)methyl-2′-amino-α-l-LNA L monomers in the same sequence context.^63^

The most densely modified Invader probes are most strongly activated for recognition of isosequential dsDNA targets (TA values = 18.0–22.5 °C, 21.5–35.0 °C, and 45.5–56.5 °C, for Invader probes with one, two, and four hotspots, respectively, Table 3), even though “TA per hotspot” values decrease with increasing levels of modification.^90^ Similar conclusions are reached by observing changes in free energy upon duplex formation. Thus, the Invader probes were found to be strongly activated for recognition of isosequential dsDNA targets (*i.e.*, Δ*G*^293^_rec_ values ≪0 kJ mol^−1^, Table 3) due to the low stability of the double-stranded probes (*i.e.*, ΔΔ*G*^293^ values between −6 kJ mol^−1^ and +11 kJ mol^−1^, relative to the corresponding dsDNA targets) and high stability of the probe-target duplexes (*i.e.*, ΔΔ*G*^293^ values between −52 kJ mol^−1^ and −6 kJ mol^−1^). Invader probes with multiple energetic hotspots displayed more favorable thermodynamics for dsDNA-recognition than single hotspot probes, and Y-modified Invader probes were more strongly activated for dsDNA-recognition than the corresponding X-modified probes (Δ*G*^293^_rec_ values more favorable by 3–31 kJ mol^−1^, Table 3). Interestingly, the X- and Y-modified Invader probes displayed more favorable thermodynamics for dsDNA-recognition than the corresponding probes based on the original^63^ 2′-*N*-(pyren-1-yl)methyl-2′-amino-α-l-LNA L monomers (Δ*G*^293^_rec_ values more favorable by 1–29 kJ mol^−1^), further highlighting their potential as second-generation monomers. Recognition of dsDNA by these Invader probes is very strongly enthalpically favored (Δ*H*_rec_ ≪ 0 kJ mol^−1^), further pointing to forced intercalation as the main driving force (stabilizing in probe-target duplexes and destabilizing in Invader probes).

**Fig. 14:**
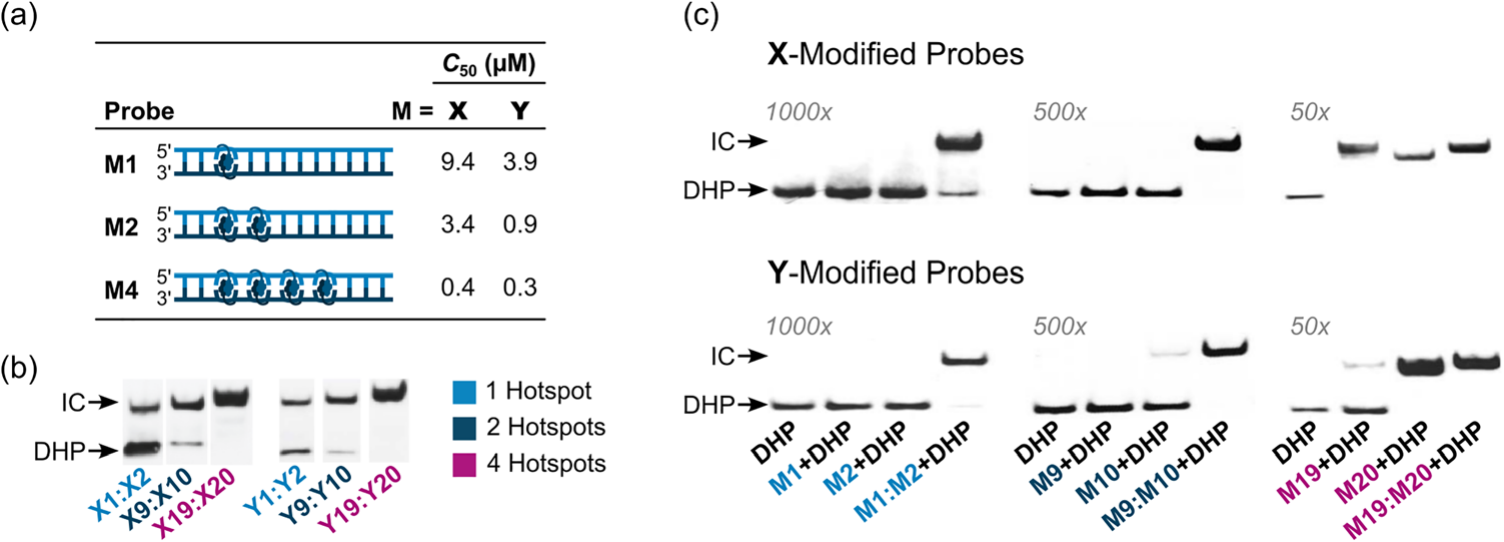
Invasion of DNA hairpins by 13-mer Invader probes featuring one, two, or four X- or Y-based energetic hotspots. (a) *C*_50_ values for X- and Y-modified Invader probes varying in hotspot density. (b) Representative electrophoretograms for dsDNA-recognition experiments in which the indicated probes were incubated at 200-fold molar excess with the corresponding DHP target. (c) DHP invasion experiments using individual Invader strands and the corresponding Invader probes at 1000-, 500-, or 50-fold molar excess, as indicated. “IC” = invasion complex. Data and figures adapted from ref. 90 with permission from the Royal Society of Chemistry.

Each of the twenty Invader probes recognized the corresponding DNA hairpin target to some degree (20–100% recognition) when incubated at 200-fold molar excess at room temperature for 17 h. Six of the probes – featuring one, two, or four consecutive hotspots of X or Y monomers – were evaluated in more detail in dose–response experiments to determine *C*_50_ values, *i.e.*, the probe concentration resulting in 50% recognition of the DNA hairpin targets (Fig. 14a). Increasing the hotspot density progressively decreased *C*_50_ values from single digit micromolar to submicromolar ranges. Y-based probes displayed lower *C*_50_ values than X-based probes in agreement with the observed Δ*G*^293^_rec_ trends.

Room temperature incubation of the DNA hairpin target with a 1000-fold excess of individual probe strands with a single X or Y incorporation, or a 500-fold excess of probe strands with two X or Y incorporations, resulted in no or merely trace formation of recognition complexes after 17 hours, whereas incubation with the corresponding Invader probes resulted in complete recognition (Fig. 14c). Conversely, room temperature incubation with a 50-fold excess of individual probe strands with four incorporations of X or Y monomers generally resulted in complete recognition of the DNA hairpin (Fig. 14c).^90^ However, only trace recognition was observed when a 2-fold excess of the individual strands with four X modifications were incubated at 37 °C, *i.e.*, conditions resulting in complete recognition of the DNA hairpin target with the corresponding Invader probe.^37^ These observations demonstrate that while the high cDNA affinity of densely modified individual Invader strands can facilitate recognition of DNA duplexes under certain conditions, the corresponding Invader probes result in more efficient recognition.

A subsequent study sought to investigate design parameters governing target recognition in other, non-TA-rich sequence contexts.^91^ Ten Cy3-labeled Invader probes were designed varying in the number of 2′-*O*-(pyren-1-yl)methyl-RNA A/C/U monomers (20–30% modified), length (14–16 base pairs), and GC-content (30–70%) (Table 4).

**Table 4:** Sequences and key parameters for Cy3-labeled Invader probes modified with 2′-*O*-(pyren-1-yl)methyl-RNA A/C/U monomers^a^

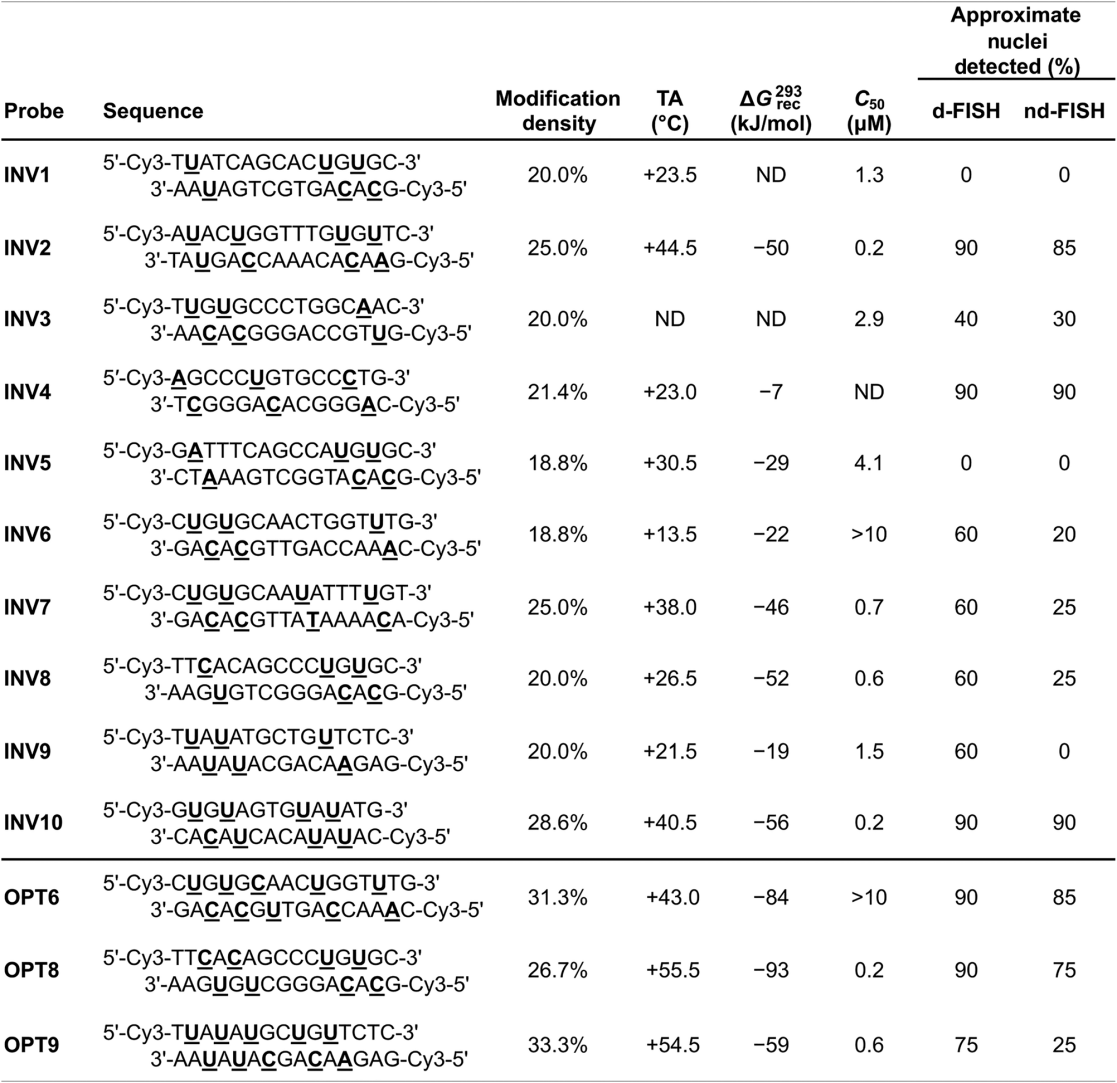

a ND = not determined.

Neither the modification density, length, or GC-content of the probes were found to be predictive of Invader probe lability as neither parameter correlated with absolute (*T*_m_ or Δ*G*^310^) or relative (Δ*T*_m_ or ΔΔ*G*^310^) measures of Invader probe stability. In contrast, the modification density correlated with Δ*T*_m_ or ΔΔ*G*^310^ values for probe-cDNA duplexes, highlighting this design parameter as a predictor of high probe-target duplex stability (Δ*T*_m_ values between +3.5 °C and +22.0 °C in this study).

As a consequence of the low stability of the probes (ΔΔ*G*_310_ values between −1 kJ mol^−1^ and +25 kJ mol^−1^) and high stability of the probe-target duplexes (ΔΔ*G*_310_ values between −33 kJ mol^−1^ and +19 kJ mol^−1^), every Invader probe was found to be thermodynamically activated for dsDNA-recognition (Δ*G*^310^_rec_ values between −56 kJ mol^−1^ and −7 kJ mol^−1^; TA values between +13.5 °C and +44.5 °C, Table 4).

Four of the ten Invader probes resulted in efficient recognition of the corresponding DNA hairpin targets when incubated for ∼15 hours at 37 °C (*C*_50_ values between 0.2 µM and 0.7 µM), five probes displayed moderately efficient recognition (*C*_50_ ∼ 1–10 µM), while one probe failed to recognize the DNA hairpin target (Table 4).

**Fig. 15:**
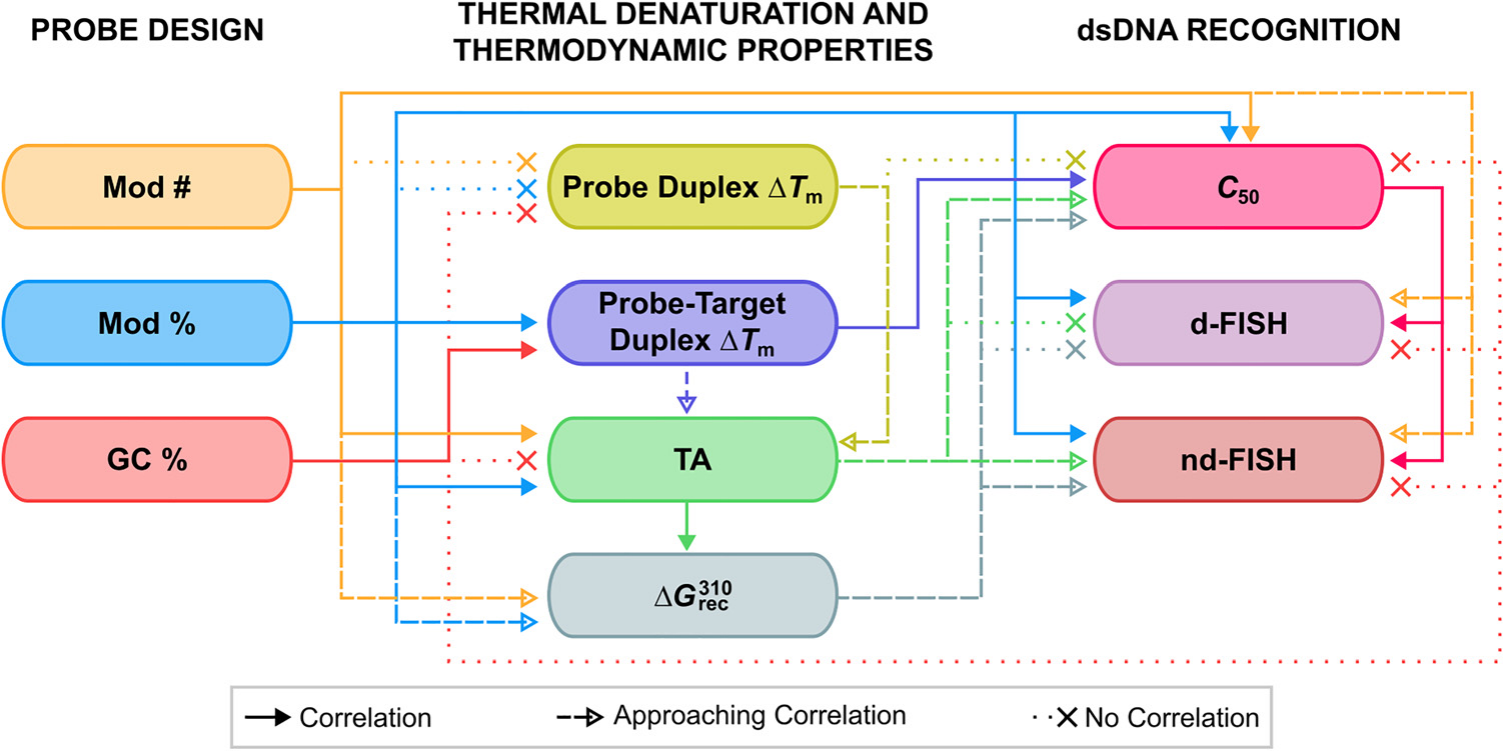
Correlations between design parameters governing recognition of dsDNA by Invader probes.

TA/Δ*G*^310^_rec_/*C*_50_ values correlated with the hotspot densities of the probes, further highlighting the latter as the key parameter for the design of efficient dsDNA-targeting Invader probes (Fig. 15). This insight was used to improve the dsDNA-targeting properties of suboptimal Invader probes. Thus, increasing the hotspot density of the probes markedly increased their TA values and lowered their *C*_50_ values to the low submicromolar range (compare INV6/8/9 and OPT6/8/9). The hotspot density was also found to be the key factor in determining the efficiency of Invader probes in fluorescence *in situ* hybridization (FISH) assays conducted under denaturing and non-denaturing conditions (*vide infra*).^91^

**Key point:** Invader probes with a density of second-generation monomers above 25% result in particularly efficient recognition of model dsDNA targets. This is due to increased cDNA affinity of individual probe strands with increasing modification densities, rather than additional destabilization of Invader probes. Recognition does not appear to be materially limited by the GC-content of a target region as recognition of model dsDNA targets with GC-contents of up to ∼70% has been demonstrated. However, since the hotspots of Invader probes are preferably constructed using pyrene-functionalized pyrimidine monomers, this calls for dsDNA target regions with a high density of 5′-pyrimidine-purine steps (Fig. 13).

## Recognition kinetics, binding specificity and invasion mechanism of second-generation Invader probes

### Recognition kinetics of second-generation Invader probes

The kinetics of dsDNA-recognition were studied using 13-mer Invader probes featuring one, two, or four consecutive X- or Y-based hotspots (for sequences, see Table 3). Invader probes with two hotspots resulted in 50% invasion of the corresponding DNA hairpin target after ∼3 hours at room temperature when used at 200-fold excess, whereas 50% recognition was observed after only 10–50 min for Invader probes with four consecutive hotspots.^90^ The corresponding pseudo-first order rate constants *k*_obs_ for the initial phases of DNA hairpin recognition of 1.1 × 10^−3^/7.5 × 10^−3^/9.7 × 10^−2^ min^−1^ and 1.4 × 10^−3^/4.3 × 10^−3^/1.5 × 10^−2^ min^−1^ were observed for the X- or Y-modified M1:M2, M9:M10 and M19:M20 Invader probes, respectively. Thus, increasing the modification density in second-generation Invader probes not only results in a greater degree of dsDNA-recognition but also faster recognition.

The extent and kinetics of Invader-mediated dsDNA-recognition are also strongly dependent on the experimental temperature.^90^ For example, a 13-mer Invader probe with two consecutive hotspots of X monomers (X9:X10, see Table 3) failed to recognize the DNA hairpin target at 8 °C, whilst resulting in ∼20%, ∼50%, and ∼80% recognition when incubated for only 10 min at 200-fold excess at room temperature, 35 °C, and 45 °C, respectively. Similar results were observed for corresponding Y-modified Invader probes. The temperature-dependent rate enhancements are mainly attributed to increased probe denaturation (*T*_m_ = 40.0 °C) rather than partial DNA hairpin denaturation (*T*_m_ = 58.5 °C). Incubation at 55 °C resulted in rapid, but incomplete DNA hairpin invasion, presumably as the invasion complex undergoes partial denaturation (*T*_m_s for probe-target duplexes were between 51.5 and 55.5 °C).^90^ The impact of incubation temperature and hotspot density on recognition kinetics was further underscored in subsequent experiments. Thus, Invader probes with three X-based energetic hotspots were shown to recognize corresponding DNA hairpin targets with *C*_50_ values of 40–50 nM when incubated at 37 °C for 2.5 hours,^92^ representing a ∼10-fold improvement relative to Invader probes with four hotspots targeting the same DNA hairpin at room temperature for 17 hours.^90^

**Fig. 16:**
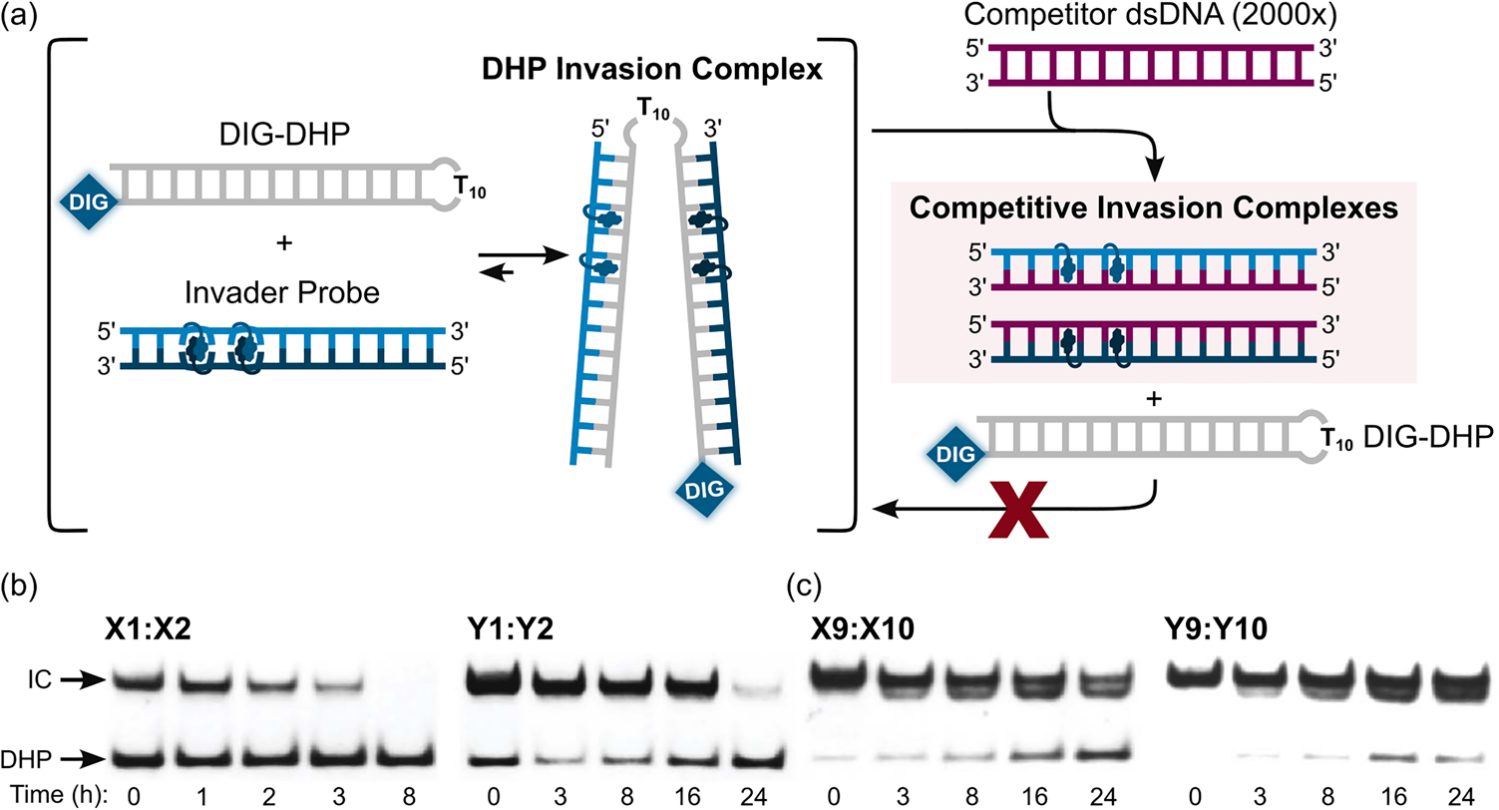
Dissociation kinetics of DHP invasion complexes. (a) Illustration of competition assay entailing pre-formed DHP-Invader invasion complexes and a large excess of competitor dsDNA target. (b) and (c) Representative electrophoretograms depicting 200-fold molar excess of Invader probe (see Table 3 for sequences) incubated with complementary DIG-DHP for 17 h at room temperature, followed by (*t* = 0 h) addition of 2000-fold molar excess of a complementary linear dsDNA target. IC = invasion complex. Electrophoretograms in (b) and (c) were reproduced from ref. 90 with permission from the Royal Society of Chemistry.

Once formed, invasion complexes entailing highly modified Invader probes are stable. Thus, 60–85% of invasion complexes – formed following incubation of a DNA hairpin target with Invader probes with two X- or Y-based hotspots – remained intact for 24 hours following treatment with a large excess of a competitor target (Fig. 16). Conversely, invasion complexes entailing single hotspot Invader probes dissociated faster (Fig. 16, only trace amounts observed after 8 h and 24 h for X1:X2 and Y1:Y2, respectively).

**Key point:** Faster invasion of dsDNA targets is observed with more extensively modified Invader probes and the resulting invasion complexes do not readily dissociate once formed. Invasion of dsDNA regions can be accelerated by raising incubation temperatures closer to or above the denaturation temperatures of the Invader probes but below the denaturation temperature of the invasion complexes.

### Invader probes bind dsDNA regions with high specificity

**Fig. 17:**
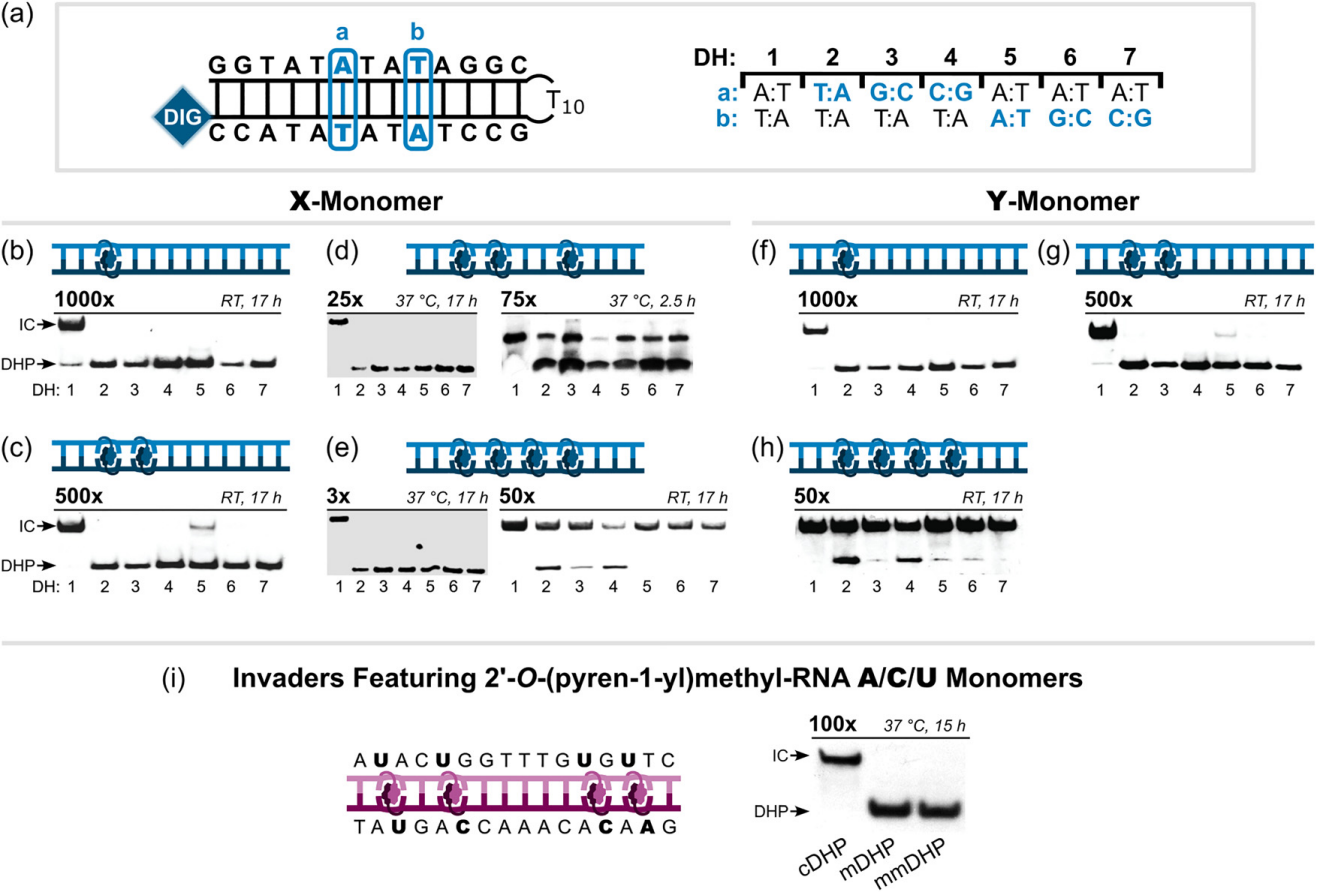
Binding specificity of second-generation Invader probes. (a) Illustration of single-mismatch hairpin targets for experiments shown in b–h where “a” and “b” indicate the position (left side of panel) and sequence (right side of panel) of the mismatches. Specificity experiments entailing (b)–(e) X-based or (f)–(h) Y-based Invader probes with one to four hotspots. (i) Specificity experiments entailing a 16-mer mixed-base Invader probe with four interspersed hotspots constructed using 2′-*O*-(pyren-1-yl)methyl-RNA A/C/U monomers incubated with complementary, single-mismatch (position 8, T:A → A:T), and doubly-mismatch (position 5, T:A → A:T, and position 12, T:A → A:T) DNA hairpin targets (cDHP, mDHP, and mmDHP, respectively). Probe excess and assay conditions are indicated above each electrophoretogram. All electrophoretograms in this figure were reproduced from ref. 36, 37, and 90 with permission from the Royal Society of Chemistry.

In addition to having prominent affinity towards complementary dsDNA regions, Invader probes display outstanding binding specificity. Early studies demonstrated that 9-mer Invader probes with one hotspot based on first- or second-generation monomers fully discriminate DNA hairpins with stems differing in sequence at merely one or two positions relative to the probes.^62^ Subsequently, 13-mer Invader probes with one or two hotspots of X or Y monomers were shown to display near-perfect discrimination of six DNA hairpins with stem regions differing only at a single central position at conditions resulting in near-complete recognition of the complementary DNA hairpins (Fig. 17b, c and f, g, respectively).^90^ The corresponding Invader probes with four X- or Y-based hotspots resulted in near-complete recognition of the six non-target DNA hairpins when incubated at 50-fold excess for 17 hours at room temperature (Fig. 17e and h, respectively.^90^ However, perfect discrimination of the non-target DNA hairpins was restored when the X-based Invader probe was used at 3-fold excess and an incubation temperature of 37 °C, *i.e.*, conditions resulting in complete recognition of the complementary DNA hairpin target (Fig. 17e).^37^ Similarly, Invader probes with three X-based hotspots in the same sequence context also resulted in perfect discrimination of these non-target hairpins when used at 25-fold (17 h incubation, Fig. 17d)^37^ or 5-fold excess (2.5 h incubation) at 37 °C,^92^ whilst resulting in complete recognition of the complementary DNA hairpin. Partial recognition of non-target DNA hairpins was observed when this Invader probe was used at a very large 75-fold excess (37 °C, 2.5 h, Fig. 17d).^92^ Remarkably, 16-mer mixed-base Invader probes with four interspersed hotspots based on 2′-*O*-(pyren-1-yl)methyl-RNA A̲/C̲/U̲ monomers were found to discriminate DNA hairpins with stems that differ in sequence at only one or two positions relative to the probes when used at 100-fold excess (37 °C, 15 h), while resulting in complete recognition of the complementary targets (Fig. 17i).^36^ Thus, high-affinity Invader probes can completely discriminate against dsDNA regions with ∼94% sequence homology, which is expected to enable single nucleotide polymorphism (SNP)-specific recognition of dsDNA target regions.

The excellent dsDNA-binding specificity of Invader probes modified with second-generation monomers contrasts the moderate binding fidelity of individual singly X- or Y-modified strands. Thus, duplexes between centrally X- or Y-modified 9-mer ONs and DNA strands with mismatched C/G/T residues opposite of the pyrene-functionalized uridine monomers displayed Δ*T*_m_ values of −13.0/−5.0/−6.5 °C and −22.0/−3.5/−12.0 °C relative to the corresponding matched duplexes, respectively, as compared to Δ*T*_m_ values of −16.5/−9.5/−17.0 °C for the corresponding unmodified ON.^74^ Interestingly, ONs with two X- or Y-monomers, placed as next-nearest neighbors (*i.e.*, a favorable probe design for Invader probes), discriminate DNA strands with mismatched A/C/G nucleotides opposite of the XAX/YAY modification cassettes with improved fidelity, *i.e.*, Δ*T*_m_ values of −24.0/−17.0/−14.0 °C and −21.5/−10.5/−13.0 °C compared to Δ*T*_m_ values of −17.0/−15.5/−9.0 for the corresponding unmodified ON.^74^

However, the high binding specificity of Invader probes likely arises primarily from stringency clamping effects, which are characteristic of structured, metastable probes.^93^ Stringency clamping increases the free-energy difference between matched and mismatched complexes by employing an oligonucleotide with a secondary structure that is less stable than the recognition complex formed with the correct target, yet more stable than the corresponding complex formed with a mismatched target. As a result, the secondary structure of the probes interferes selectively with mismatched binding, yielding higher specificity than unstructured oligonucleotides.

**Fig. 18:**
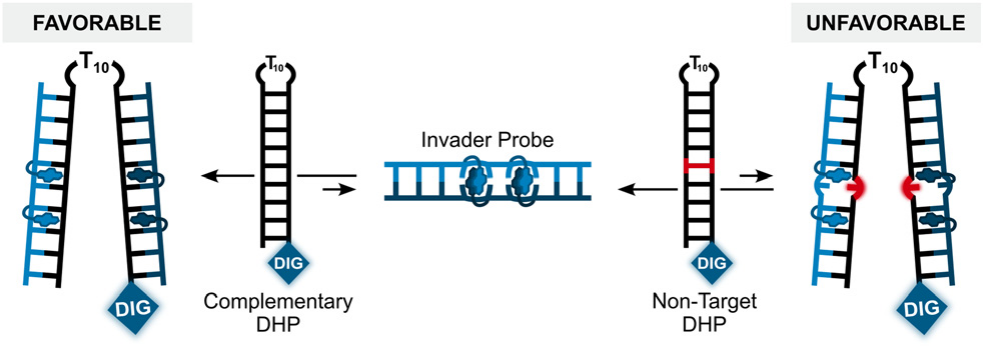
Stringency clamping effects underlying the excellent binding specificity of Invader probes. The non-target DHP represents a DHP with a fully base-paired stem region but differing in sequence at one position (red) relative to the Invader probe.

Established examples of stringency clamping include hairpin-based probes (*e.g.*, molecular beacons) in duplex formation and imperfect duplexes in triplex recognition.^93^ By analogy, productive binding of Invader probes to non-target dsDNA would require both denaturation of the double-stranded probe and target region and formation of two mismatched duplexes within the invasion complex. The double-stranded Invader probes are presumed to be less stable than the ternary invasion complexes formed with the correct DNA hairpin target, but more stable than complexes formed with non-target sequences containing two destabilized mismatched duplexes (Fig. 18). Consequently, Invader probes function as stringency clamps by selectively disfavoring non-target binding and amplifying the thermodynamic discrimination between target and non-target interactions.

**Key point:** Invader probes based on second-generation monomers exhibit exceptional binding specificity due to stringency clamping effects and can discriminate against dsDNA-regions with ∼94% sequence homology.

### Proposed mechanism for Invader probe-mediated dsDNA-recognition

**Fig. 19:**
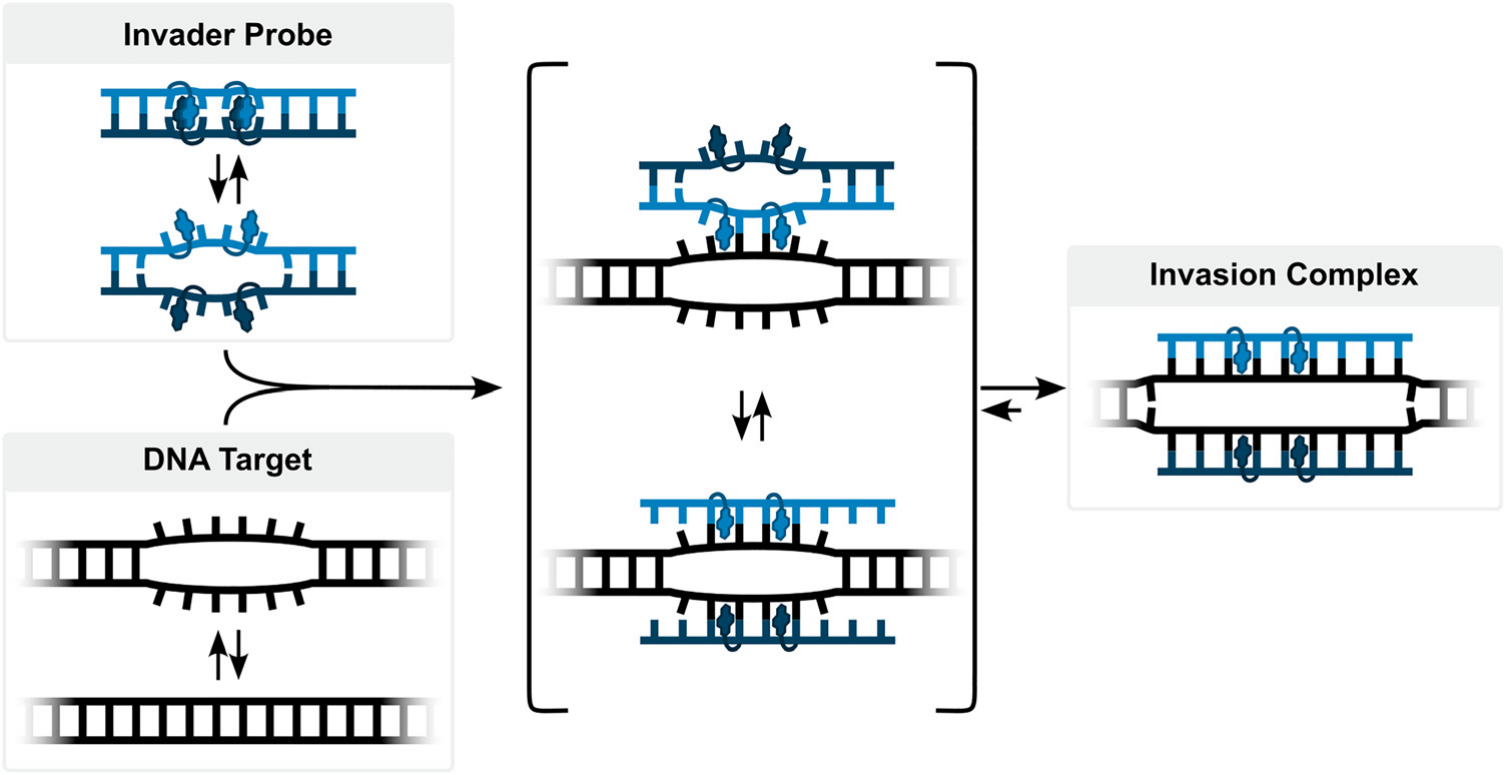
Proposed double-duplex invasion mechanism for recognition of complementary dsDNA regions by Invader probes.

While no definitive proof for the mechanism of Invader-mediated dsDNA-recognition has been obtained, we stipulate that densely modified Invader probes are partially denatured and significantly perturbed, thereby enabling the pyrene moieties to initially interact non-specifically with – and intercalate into – particularly accessible regions of DNA duplexes. This may initiate nucleation and unzipping of Watson–Crick base pairs, ultimately resulting in recognition of complementary dsDNA regions *via* a double-duplex invasion mechanism that resembles the mechanism proposed for pseudo-complementary PNA (Fig. 19).^31^

## Comparison of second-generation Invader probes with other DNA-targeting probes

Having established the main design guidelines for second-generation Invader probes, we set out to compare their dsDNA-recognition characteristics with other strand-invading probes, including INAs and single-stranded ^Ser^γPNAs, ^MP^γPNAs, and partially or fully LNA-modified ONs.^36,37,92^

**Fig. 20:**
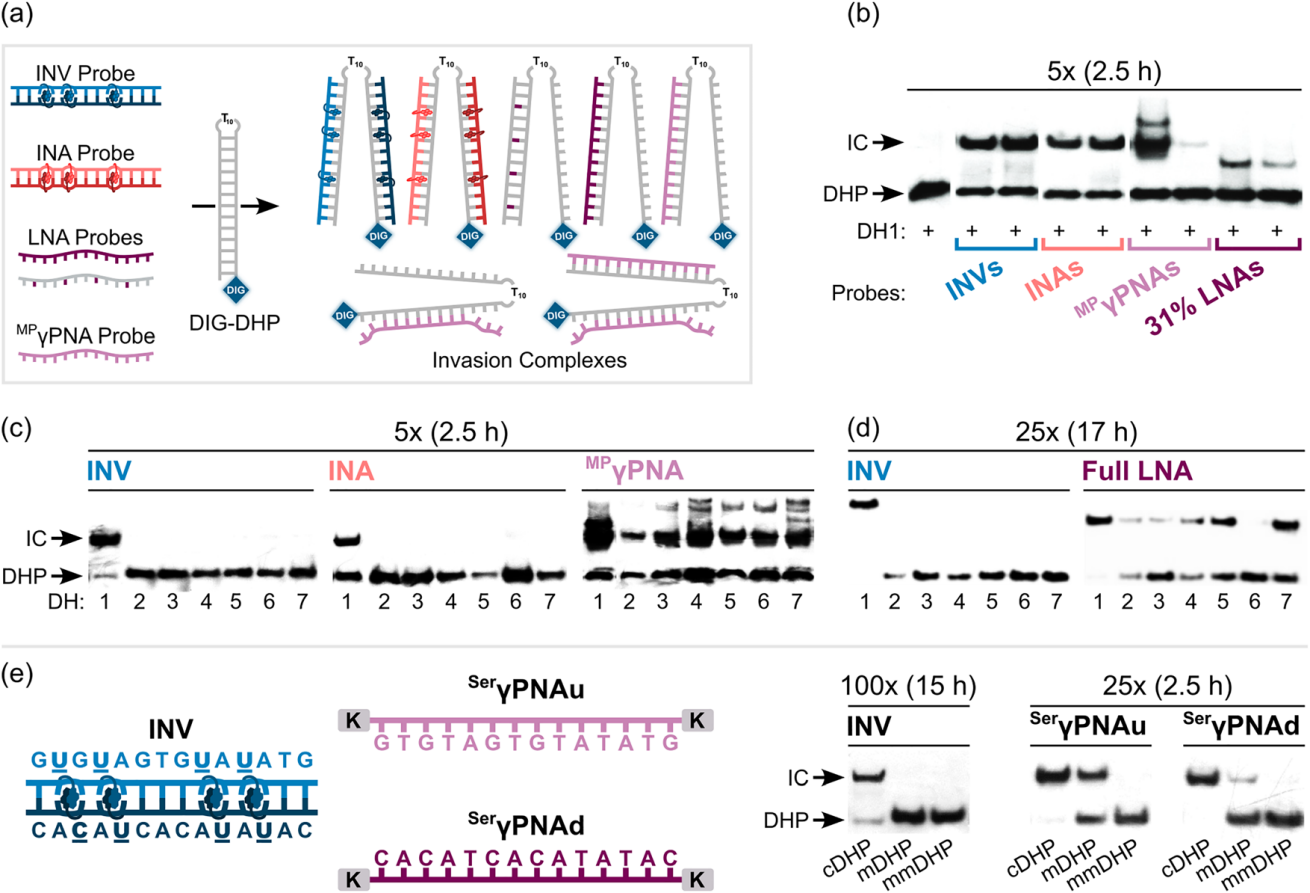
Comparison of dsDNA-invasion characteristics of Invader, INA, ^MP^γPNA, LNA, and ^Ser^γPNA probes. (a) Illustration of the DIG-DHP invasion assay using various probes. (b) Representative gel electrophoretogram from dsDNA-invasion experiments in which DH1 (complementary target to the probes) was incubated with the indicated probe type. (c) and (d) Representative gel electrophoretograms from binding specificity experiments. (e) Sequences of 14-mer Invader and ^Ser^γPNA probes (K = lysine), and invasion assay results with complementary DHP (cDHP), singly mismatch (m), or doubly mismatch (mm) DHP targets. Probe excess and incubation times are as listed; all incubations were performed at 37 °C. For structures of modifications, see Fig. 2. See Fig. 17a for sequences of DH1–DH7. All electrophoretograms in this figure were reproduced from ref. 37 and 92 with permission from the Royal Society of Chemistry.

In a first comparison study,^92^ two 13-mer Invader probes with three X-based hotspots recognized the corresponding DNA hairpin target with similar efficiency as the equivalent INA probes (*C*_50_ = 30–50 nM, 37 °C, 2.5 h incubation), whereas ∼30% LNA-modified single-stranded ONs only resulted in moderate dsDNA-recognition despite the affinity-enhancing properties of LNA monomers (Δ*T*_m_ values of ∼17 °C were observed for duplexes with cDNA) (Fig. 20; for monomer structures see Fig. 2). Two ^MP^γPNA probes were found to form very stable duplexes with cDNA (Δ*T*_m_ > 40 °C), but just one resulted in moderate recognition of the DNA hairpin (*C*_50_ ∼ 250 nM), whereas the other only resulted in trace recognition (Fig. 20). Both ^MP^γPNA probes displayed a proclivity for secondary structure formation in the AT-rich, partially self-complementary sequence context studied (see Table 3 for sequence context) as concentration-dependent *T*_m_ trends – consistent with dimer formation – were observed in the absence of cDNA targets. Formation of stable secondary structures is commonly observed for single-stranded probes with affinity-increasing modifications, which may interfere with dsDNA-recognition.^41^ Moreover, the dsDNA-binding ^MP^γPNA probe was found to form a partial duplex with the isosequential (*i.e.*, non-target) arm of the DNA hairpin, indicating potential specificity challenges.

The double-stranded Invader and INA probes resulted in slower but far more specific dsDNA-recognition than the ^MP^γPNA probe. Thus, when used at 37 °C and 5-fold excess relative to the DNA hairpin target, the ^MP^γPNA probe resulted in 50% invasion in less than 10 min, while it took 37–68 min and 55–75 min, respectively for 50% invasion to be reached with the equivalent triple hotspot Invader and INA probes. This likely reflects the different recognition mechanisms of ^MP^γPNA *vis-à-vis* Invader/INA probes, *i.e.*, single- *vs.* double-duplex invasion, respectively, with the latter requiring partial denaturation of the double-stranded probes to reveal nucleation sites that can initiate formation of invasion complexes with the dsDNA target (Fig. 19). Invader and INA probes resulted in complete discrimination of DNA hairpins with stems differing in sequence at one of thirteen positions *vis-à-vis* the probes, whereas the ^MP^γPNA resulted in minimal discrimination of these targets (Fig. 20c), which highlights the advantage of the stringency clamping effect displayed by double-stranded probes.

A subsequent comparison study^37^ – using the same DNA hairpins – revealed that the corresponding, fully modified LNA probes display similar dsDNA-recognition characteristics as the ^MP^γPNA probes; one LNA probe resulted in moderate recognition (*C*_50_ ∼ 300 nM, 37 °C, 17 h incubation), while the other LNA probe only resulted in trace recognition. In contrast, Invader probes with three or four energetic hotspots displayed *C*_50_ values of ∼85 nM and ∼35 nM, respectively. The single-stranded LNA probe displayed poor discrimination of the non-target DNA hairpins when used at 25-fold excess, whereas the Invader probes displayed complete discrimination of these hairpins (Fig. 20d).

To determine if dsDNA-regions with significant self-complementarity are particularly challenging targets for single-stranded γPNA probes, we set out to study the dsDNA-invasion properties of ^Ser^γPNA and Invader probes targeting DNA hairpins with low levels of self-complementarity.^36^ Invader probes resulted in efficient, highly specific, but comparatively slow invasion of DNA hairpin targets, featuring complementary 14–16 base-paired stems and varying GC-content levels. Invasion was equally efficient but faster with the single-stranded ^Ser^γPNA probes. Thus, three of six ^Ser^γPNA probes and two of three Invader probes recognized the corresponding DNA hairpin targets with *C*_50_ values between 0.12–0.35 µM (2.5 h incubation, 37 °C) and 0.16–0.21 µM (15 h incubation, 37 °C), respectively. Curiously, the denaturation profiles of three of six ^Ser^γPNA probes in absence of cDNA were consistent with the formation of stable secondary structures in these sequence contexts. Additionally, at least two ^Ser^γPNA probes displayed signs of aggregation and low solubility. Furthermore, DNA hairpins differing in sequence at one of 14–16 positions were poorly discriminated by ^Ser^γPNA probes, whereas the corresponding Invader probes resulted in complete discrimination of these hairpins (Fig. 20e), once again underlining the advantageous impact of the stringency clamping effect that double-stranded probes exhibit. The lower binding specificty of the ^Ser^γPNA probes also manifested itself in less specific labelling of corresponding chromosomal DNA regions (*vide infra*).

**Key point:** Optimized Invader probes allow for equally efficient recognition of mixed-sequence dsDNA targets with high levels of self-complementarity as corresponding INA probes, whereas single-stranded ^MP^γPNA and fully modified LNA probes result in less efficient and less specific, but faster recognition. Invader probes result in equally efficient, substantially more specific, but slower recognition than single-stranded ^Ser^γPNA probes when targeting mixed-sequence dsDNA regions with low levels of self-complementarity.

## Applications of Invader probes

Having demonstrated the effectiveness of second-generation Invaders probes for recognition of model mixed-sequence dsDNA targets using fluorescence and PAGE-based assays, we set out to establish proof-of-concept for Invader-mediated recognition of biological dsDNA targets.

**Fig. 21:**
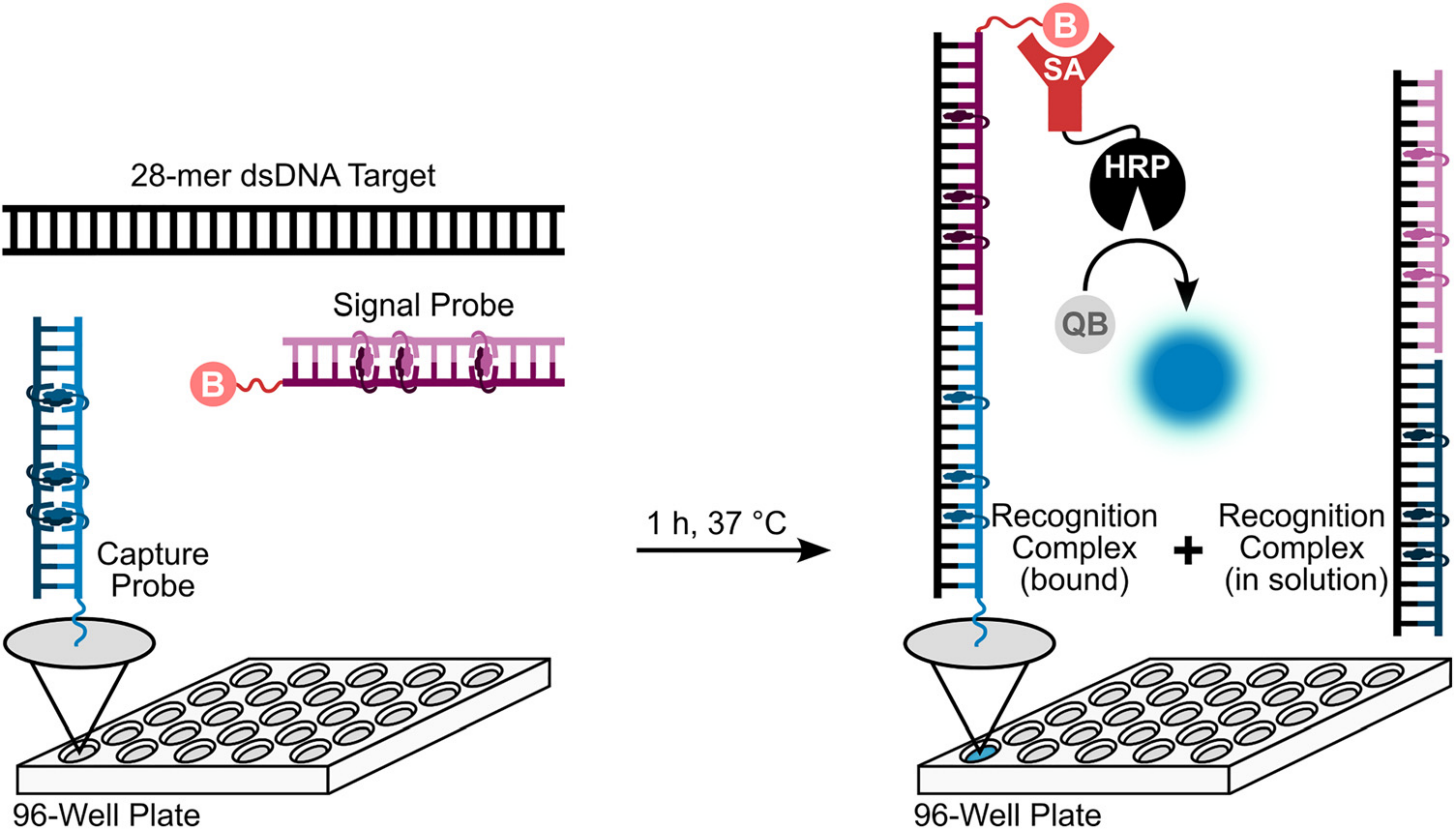
Sandwich-based assay for detection of dsDNA targets specific to food pathogens.

In a first example,^94^ we developed a 96-well plate sandwich assay based on Invader capture and signalling probes designed to recognize mixed-sequence dsDNA regions specific to three foodborne pathogens, *i.e.*, *Salmonella enterica*, *Campylobacter jejuni*, and *Escherichia coli*. Amine-terminated Invader capture probes were linked to the surfaces of 96-well plates and co-incubated with biotinylated Invader signalling probes and pathogen-specific dsDNA. Successful Invader-mediated recognition of the corresponding dsDNA targets was expected to result in the formation of two ternary complexes, *i.e.*, one in solution and one bound to the well (Fig. 21). Bound recognition complexes were detected using horseradish peroxidase (HRP)-conjugated streptavidin and subsequent incubation with an HRP substrate that forms a fluorescent product for convenient signal readout. Three sets of 14-mer Invader signalling and capture probes – each featuring three X-based hotspots – were designed to span the 28-mer pathogen-specific mixed-sequence dsDNA targets with GC-contents between 32% and 50%. As expected, the Invader capture and signalling probes were found to be relatively labile (Δ*T*_m_ values between −5.0 °C and +4.0 °C, compared to the corresponding unmodified DNA duplexes), while individual probe strands displayed increased cDNA affinity (Δ*T*_m_ values between +9.0 °C and +19.0 °C) resulting in favorable activation for dsDNA-recognition (TA values between 21.0 °C and 40.0 °C). Incubation of Invader capture and signalling probes with the corresponding dsDNA targets (1 h, 37 °C) resulted in dose-dependent signal formation with a target sensitivity of 20–55 pM. In contrast, very weak signals were observed when signalling/capture probes designed for one species (*e.g.*, *E. coli*) were incubated with dsDNA targets specific to another species (*e.g.*, *Salmonella*), further underscoring the high specificity of Invader-mediated dsDNA-recognition.

**Fig. 22:**
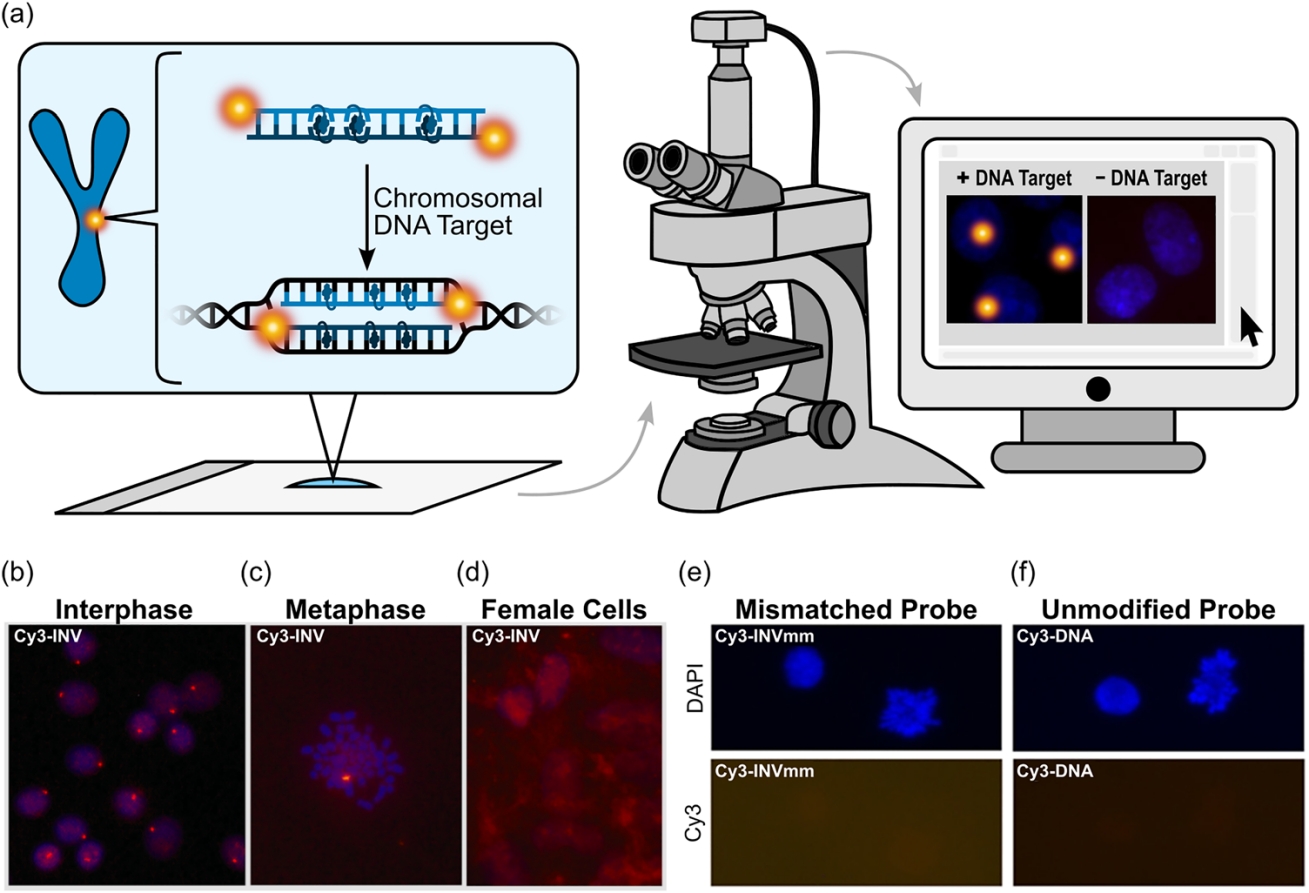
Chromosomal detection in nd-FISH assays. (a) Illustration of assay. Representative fluorescence microscopy images from nd-FISH experiments using a target-specific Invader probe (Cy3-INV, 5′-Cy3-**A**GCCC**U**GTGCC**C**TG:3′-T**C**GGGA**C**ACGGG**A**C-Cy3) incubated with fixed nuclei from a male bovine cell line captured in (b) interphase and (c) metaphase, or (d) fixed nuclei from a female bovine cell line, or using (e) non-targeting Invader probe (Cy3-INVmm – sequence differing at three positions relative to target, 5′-Cy3**-A**GC*G*C**U**G*A*G*G*C**C**TG:3′-T**C**G*C*GA**C***T*C*C*GG**A**C-Cy3), or (f) an unmodified DNA duplex with the same sequence as Cy3-INV (Cy3-DNA) incubated with fixed nuclei from the male bovine cell line under nd-FISH conditions. Images in panels (b)–(f) were reproduced from ref. 36, 89, and 91 from the Royal Society of Chemistry and MDPI.

In a series of subsequent proof-of-principle studies,^89–91^ we set out to utilize X-based Invader probes for recognition of chromosomal DNA in non-denaturing fluorescence *in situ* hybridization (nd-FISH) experiments (Fig. 22a). Unlike conventional FISH assays, which require chemical and/or heat-induced denaturation of chromosomal DNA,^95^ nd-FISH assays strive to map chromosomal loci under mild conditions. Towards this end, we selected a unique site within the highly repeated DYZ-1 satellite region (∼6 × 10^4^ repeats) on the bovine (*Bos taurus*) Y chromosome as a model target. An initial set of four Cy3-labeled Invader probes – varying in length (11–14 base pairs) and number (2–3) and location of hotspots – were designed against the same target region in this site. As expected, the Invader probes were relatively labile (Δ*T*_m_ values between −3.5 °C and +1.0 °C relative to the corresponding unmodified DNA duplexes), while the individual strands formed stable duplexes with cDNA (Δ*T*_m_ values between +8.5 °C and +15.0 °C), resulting in a prominent driving force for dsDNA-recognition (TA values between +23.0 °C and +26.5 °C). The Cy3-labeled Invader probes were incubated with fixed interphase nuclei from a male bovine kidney cell line under non-denaturing conditions (3 h, 38.5 °C, 10 mM Tris-Cl, pH 8.0 and 1 mM EDTA), producing a single, punctate fluorescent signal that localized to the heterochromatic region, consistent with the expected localization of the DYZ-1 satellite target (Fig. 22b). One or two localized signals per nucleus – consistent with post- and premitotic nuclei – were observed when the probes were incubated with nuclei captured in metaphase (Fig. 22c). Each probe resulted in robust signals, with little variation between the probes. The high labeling coverage (∼90%), *i.e.*, the proportion of nuclei with localized signals, is noteworthy considering the high GC content of the target region (∼71% GC).

Pre-treatment of nuclei with RNase A or proteinase K prior to incubation with the Invader probes, resulted in similar signals as in nuclei without pretreatment, while nuclei pre-treated with DNase I were devoid of localized signals, pointing to DNA as the target of the Invader probes.^89^ Additionally, an absence of localized signals were observed when (i) fixed nuclei from a female bovine fibroblast cell line were incubated with Y-chromosome-specific Invader probes (Fig. 22d), or (ii) when Invader probes differing in sequence at three positions *vis-à-vis* the DYZ-1 target site were incubated with fixed nuclei from the male bovine kidney cell line (Fig. 22e). A Cy3-labeled but otherwise unmodified control of the Invader probes (Cy3-DNA) also failed to produce a signal (Fig. 22f). In concert, these results demonstrate that Invader-mediated recognition of mixed-sequence chromosomal DNA targets proceeds efficiently and in a highly specific manner.

**Fig. 23:**
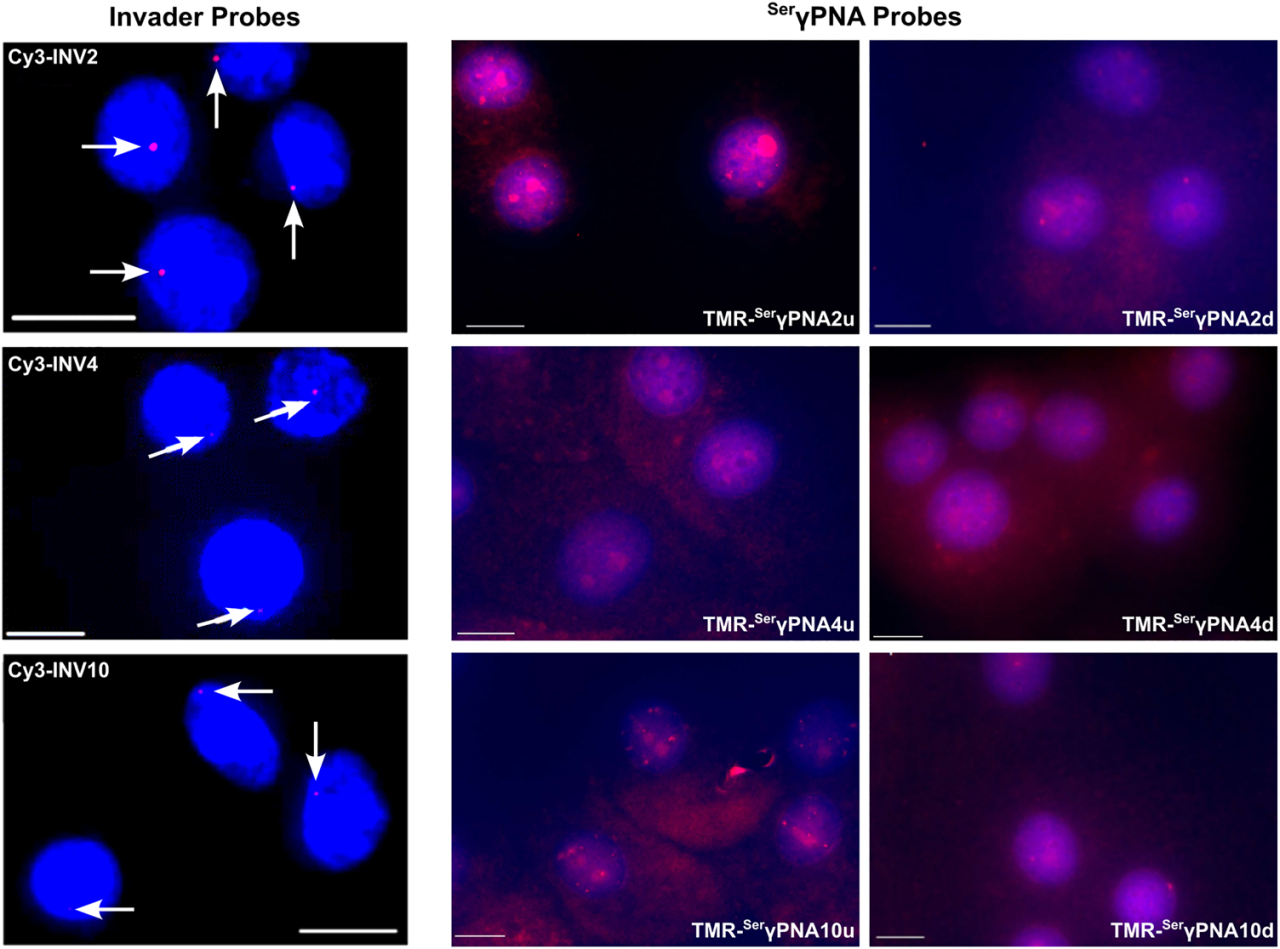
Representative fluorescence microscopy images from nd-FISH experiments in which target-specific Invader probes (Cy3-INV2, Cy3-INV4, and Cy3-INV10) and the corresponding ^Ser^γPNA probes (TMR-^Ser^γPNA2u, TMR-^Ser^γPNA2d, TMR-^Ser^γPNA4u, TMR-^Ser^γPNA4d, TMR-^Ser^γPNA10u, and TMR-^Ser^γPNA10d) were incubated with fixed nucleic from a male bovine kidney cell line. Reproduced from ref. 36 with permission from the Royal Society of Chemistry.

In a subsequent study,^91^ ten Invader probes based on 2′-*O*-(pyren-1-yl)methyl-RNA A̲/C̲/U̲ monomers – varying in length (14–16 base pairs), hotspot content (20–30%), and GC-content (30–70%) – were designed to target a broader range of complementary sequences within the DYZ-1 region (Table 4). As discussed earlier, each of these Invader probes was found to be prominently activated for dsDNA-recognition, with all but two resulting in moderate to efficient recognition of the corresponding DNA hairpin targets (*C*_50_ values between 0.2 µM and 4.1 µM, Table 4). These Invader probes were incubated with fixed interphase nuclei from the male bovine kidney cell line under denaturing (d) or nd-FISH conditions. The d-FISH assay was expected to yield information about the maximal recognition capacity of each probe, as kinetic barriers to accessing the chromosomal DNA target regions are minimized by the high incubation temperatures (5 min, 80 °C). Conversely, the nd-FISH experiments were designed to reveal if a probe can recognize the corresponding choromosomal DNA target at more physiologically relevant conditions. Three probes were found to recognize their DNA targets with excellent efficiency in the d-FISH assays (*i.e.*, ∼90% of the analyzed interphase nuclei displaying a single, intense, punctate signal against a low level of background), five probes displayed single punctate signals in 40–60% of the analyzed nuclei, while two probes failed to yield acceptable signal profiles (Table 4). The Invader probes largely retained their signaling characteristics under nd-FISH conditions (Table 4). Thus, three probes yielded single, intense, and punctate signals against a low background in 85–90% of the analyzed nuclei (Fig. 23), four probes displayed moderately intense signals in 20–30% of the nuclei, while three probes did not produce discernable signals (Table 4). In other words, most of the studied Invader probes resulted in acceptable-to-excellent recognition of chromosomal DNA targets under d-FISH and nd-FISH conditions.

A Spearman's rank-order correlation analysis revealed that signaling performance in d-FISH and nd-FISH assays correlated with the hotspot density of the probes and *C*_50_ values from the DHP assays (Fig. 15). Moreover, correlations approaching statistical significance were observed between nd-FISH (but not d-FISH) signaling performance and TA and Δ*G*^310^_rec_ values, indicating that these metrics have predictive value for nd-FISH, but not d-FISH, assay performance.

Having identified hotspot density as the key parameter for successful dsDNA-recognition, the hotspot density in three Invader probes displaying poor-to-moderate signaling characteristics under nd-FISH conditions (INV6, INV8 and INV9) was increased from ∼20% to ∼30% (OPT6, OPT8, OPT9) (Table 4). As predicted, the optimized probes displayed more prominent driving forces for recognition of dsDNA targets than the parent probes (TA values between 43.0–55.5 °C *vs.* 13.5–26.5 °C, respectively; Δ*G*^310^_rec_ values between −93 kJ mol^−1^ and −59 kJ mol^−1^*vs.* between −52 kJ mol^−1^ and −19 kJ mol^−1^, respectively) and lower *C*_50_ values, whilst still displaying excellent binding spcificity. In agreement with these trends, improved signaling characteristics, relative to the parent probes, were observed for the optimized probes. Thus, 75–90% of the nuclei displayed prominent, single, and punctate signals when optimized probes were used under d-FISH conditions compared to ∼60% of nuclei when parent probes were used (Table 4). Along similar lines, ∼85%, ∼75% and ∼25% of the nuclei displayed high-quality signals when the three optimized probes were used under nd-FISH conditions, respectively, as compared to 0–25% with the parent probes (Table 4). This indicates that increasing the hotspot density is an effective strategy to improve the signalling characteristics of Invader probes.

The nd-FISH signalling characteristics of six TAMRA-labelled ^Ser^γPNA probes, targeting the sense or antisense strand of the same regions as the three most promsing Cy3-labelled Invader probes, were determined next.^36^ Two of the ^Ser^γPNA probes resulted in the formation of single punctate signals in ∼70% of interphase nuclei from the male bovine kidney cell line but with a stronger non-specific background than with the Invader probes, which produced single punctate signals in >85% of the interphase nuclei (Fig. 23). The remaining ^Ser^γPNA probes resulted in no or minimal target-specific recognition; instead, multiple signals and/or intense diffuse background were observed. Thus, despite the two probe chemistries displaying comparable binding affinities against the DNA hairpin targets (see the section “*Comparison of second-generation invader probes with other DNA-targeting probes*”), considerable differences were observed in the nd-FISH experiments.

**Fig. 24:**
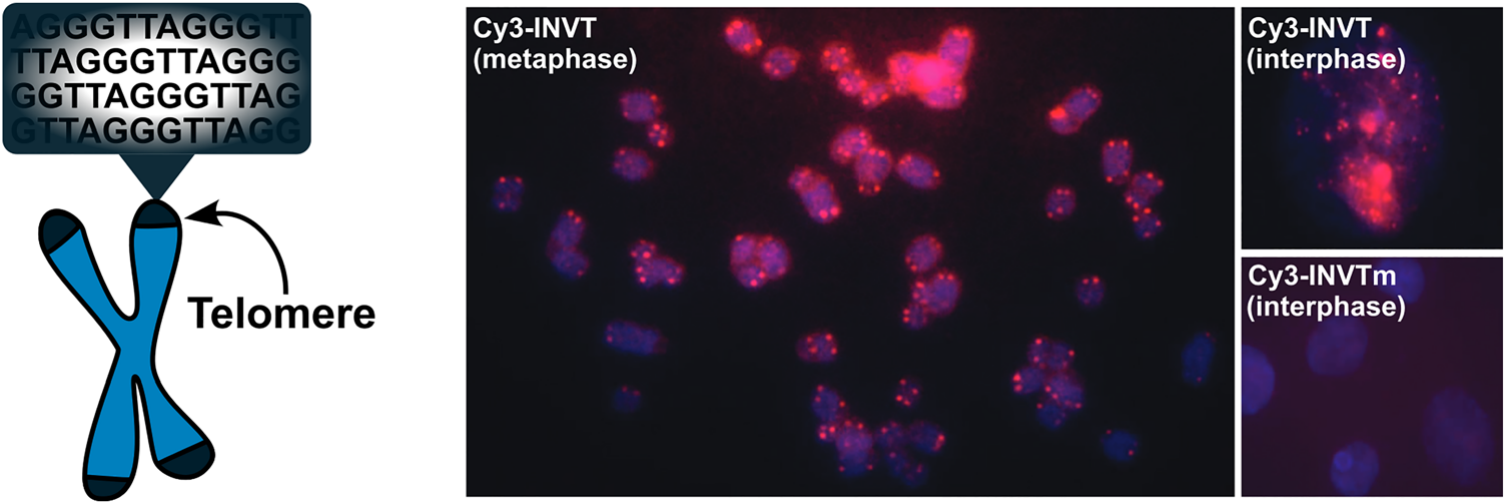
Illustration of telomere target and representative fluorescence microscopy images from nd-FISH experiments in which target-specific Invader probe (Cy3-INVT) and single-mismatched Invader probe (Cy3-INVTm) were incubated with isolated nuclei in metaphase or interphase spreads. Reproduced from ref. 92 with permission from the Royal Society of Chemistry.

Invader probes have also been evaluated for their ability to target telomeric DNA.^92^ Telomeres are guanosine-rich tandem repeats (5′-TTAGGG-3′, in vertebrates) capping chromosome termini and have central roles in aging, age-associated diseases, and cancer (Fig. 24).^96,97^ Accordingly, there has been considerable interest in developing fluorescence-based diagnostic tools that enable detection of telomeres.^7,98–102^ Towards this end, a 16-mer Cy3-labeled Invader probe with three X-based hotspots was designed. The probe displayed the expected thermal denaturation properties, *i.e.*, the probe duplex was less stable than the corresponding duplexes between individual probe strands and cDNA, although the stability differences were less pronounced (TA = 19.5 °C). Nonetheless, incubation of this Invader probe with isolated nuclei from a male bovine kidney cell line, produced pairs of bright foci on the termini of most metaphasic chromosomes in nd-FISH experiments (Fig. 24). In contrast, a corresponding 16-mer Invader probe differing in sequence at one position relative to the telomeric repeat, did not not result in signal formation (Fig. 24).^92^

**Key point:** Appropriately designed Invader probes have found utility in diagnostic assays and enable highly specific and sequence-unrestricted recognition of chromosomal DNA targets at denaturing and non-denaturing conditions.

## Conclusion and future outlook

What began as a curiosity-driven study with no clear application beyond the pursuit of improved LNA analogues (*i.e.*, synthesis and biophysical characterization of ONs modified with N2′-functionalized 2′-amino-α-l-LNA monomers), led to an unexpected discovery: DNA duplexes with +1 interstrand zippers of intercalator-functionalized nucleotides are labile, while individual strands display remarkable affinity for complementary DNA. This serendipitous finding evolved into a multi-year effort that yielded a new and distinctive class of strand-invading probes capable of sequence-unrestricted recognition of dsDNA target regions – the Invader probes. Much of our work has focused on the chemical refinement of Invader probes through optimization of the monomers^76–78,82,103^ and probe architectures,^35–38,104–110^ which will be the subject of a separate account. In the process, more than ten graduate students and fifteen undergraduate students have received training in synthetic organic chemistry, nucleic acid chemistry, and molecular biology – most now contributing their expertise in the pharmaceutical industry.

Although key evidence already supports the utility of Invader probes – most notably their ability to target specific chromosomal DNA regions under nd-FISH conditions – much remains to be explored. Demonstrating efficient nuclear uptake and gene knockdown in cell culture would provide important proof to establish Invader probes as a solution to the long-standing challenge of robust and sequence-unrestricted dsDNA-recognition. Unpublished studies – using sub-optimal, sparsely modified, first-generation Invader probes – have indicated that inhibition of *in vitro* transcription and gene knockdown in luciferase reporter assays in cell cultures is possible, suggesting that the concept is viable and primed for further exploration using optimally designed, next-generation Invader probes in collaboration with domain experts. Other applications of Invader probes seem possible including their use as PCR blockers for enrichment of rare mutants, artificial restriction enzymes, and non-protein-based gene editing constructs.

In summary, Invader probes reveal that directed intercalation can be harnessed to overcome the energetic penalty of duplex invasion to achieve sequence-unrestricted access to double-stranded DNA without reliance on proteins.

## Conflicts of interest

PJH is an inventor on patents pertaining to Invader probes, which have been issued to the University Idaho.

## Data Availability

No primary research results, software or code have been included and no new data were generated or analysed as part of this review.
